# Single-cell delineation of strain-specific HIV-1 Vif activities using dual reporter sensor cells and live cell imaging

**DOI:** 10.1128/jvi.01579-24

**Published:** 2025-02-25

**Authors:** Jorge F. Guerrero, Laraine L. Zimdars, James W. Bruce, Jordan T. Becker, Edward L. Evans, Soroosh Torabi, Rob Striker, Scott M. Berry, Nathan M. Sherer

**Affiliations:** 1 McArdle Laboratory for Cancer Research (Department of Oncology), University of Wisconsin-Madison 219455 , Madison, Wisconsin, USA; 2 Institute for Molecular Virology, University of Wisconsin-Madison 70033 , Madison, Wisconsin, USA; 3 Department of Mechanical Engineering, University of Kentucky 4530 , Lexington, Kentucky, USA; 4 Department of Medicine, University of Wisconsin-Madison 200889 , Madison, Wisconsin, USA; University Hospital Tübingen, Tübingen, Germany

**Keywords:** HIV, genome diversification, reporter cell, Vif, APOBEC3G, cell cycle, live cell imaging, image analysis

## Abstract

Human immunodeficiency virus type 1 (HIV-1) genome diversification is a key determinant of viral evolution and the pathogenesis of HIV/AIDS. Antiretroviral therapy is non-curative, and in the context of monitoring the latent reservoir, precision tools are needed to detect and enumerate HIV-1 genomes as well as to assess their heterogeneity, replication potential, and predict responses to therapy. Current sequencing-based methodologies are often unable to confirm intact genomes and most cell-based reporters provide limited information pertaining to viral fitness. In this study, we describe dual reporter sensor cells (DRSCs), an imaging-based reporter system designed to detect HIV-1 infection and measure several independent attributes of the virus in a single-cell high-content assay. We show that the DRSC assay can be used to measure infection, viral gene activation kinetics, and quantify viral circumvention of host antiviral responses. Using the DRSCs, we confirmed markedly different functional heterogeneity for *vif* alleles derived from diverse HIV-1 strains and subtypes affecting both rates of APOBEC3G degradation and the cell cycle. Furthermore, the assay allowed for the delineation of virus co-receptor preference (X4- vs R5-tropism) and visualization of virion assembly. Overall, our study illustrates proof-of-principle for a multivariate imaging-based cell-based system capable of detecting HIV-1 and studying viral genetic variability with greater data richness relative to prior available modalities.

**IMPORTANCE:**

Human immunodeficiency virus type 1 (HIV-1) is highly heterogeneous and constantly mutating. These changes drive immune evasion and can cause treatment efforts to fail. Here, we describe the “dual reporter sensor cell” (DRSC) assay; a novel imaging-based approach that allows for the detection of HIV-1 infection coupled with a multivariate definition of several independent phenotypic aspects of viral genome activity in a single integrated assay. We validate the DRSC system by studying lab-adapted and patient isolate-derived versions of the viral Vif accessory protein, confirming marked differences in the capacity of diverse *vif* alleles to mediate downregulation of antiviral APOBEC3G proteins and dysregulate the cell cycle.

## INTRODUCTION

Genetic variability is a product of evolution common to all viruses. Retroviruses such as the human immunodeficiency virus type 1 (HIV-1) and other RNA and DNA viruses exhibit high genome heterogeneity due to mechanisms of gene variation including mutation, recombination, or for some viruses (e.g., influenza virus) genome segment reassortment (1–5). Viral evolution in the face of selective forces can yield HIV-1 founder strains with altered pathogenicity due to changes in transmission rate (6–9) or viral fitness (10, 11). Rapid genome diversification is also the basis for viral evasion of the host immune system and resistance to antiviral therapies (12–18).

HIV-1 genome diversification is driven by both error-prone reverse transcription and genome recombination. Over recent decades, the major circulating clade (group M) has diverged into subtypes including A1, A2, A3, A4, B, C, D, F1, F2, G, H, J, K (9). HIV-1 subtype B is the most prevalent in the West and has received the lion’s share of HIV-1 research. However**,** subtype C is endemic to Sub-Saharan Africa and represents 47% of total HIV-1 infections worldwide (19). Emerging recombinant strains such as AE and AG are also of increasing concern (20). Interestingly, HIV-1 utilizes two coreceptors, CXCR4 and CCR5, to initiate infection along with its main receptor CD4. The coreceptor is necessary for infection, but different strains of HIV-1 have a bias toward using one coreceptor over the other. Viruses that primarily use the CXCR4 coreceptor are termed X4-tropic, CCR5-biased viruses are X5-tropic, and dual-tropic viral strains also exist which can utilize either coreceptor for infection (21).

Even within single subtypes, HIV-1 can exhibit significant variability among hosts (up to 20% sequence divergence at the nucleotide level) (22, 23), with these differences of potential relevance to viral persistence and/or resistance to therapy. For example, HIV-1 Envelope (24–26), Capsid, Reverse Transcriptase, and Integrase each exhibit mutations that perturb the efficacy of pharmacological and antibody treatment options (27–29). Similarly, the regulatory and accessory proteins Rev, Tat, Vif, Vpr, Vpu, and Nef can exhibit functional variability based on gene heterogeneity thought to impact pathogenic outcomes (30–45).

To track viral evolution and study the latent reservoir, there is a dire need for precision tools to detect, enumerate, and characterize HIV-1 genomes and their diversification. Multiple techniques are currently available for these purposes, of which the most prevalent and cost-effective are polymerase chain reaction (PCR)-based genome amplification methods coupled to sequencing (46, 47). While sensitive, a limitation to these strategies is that sequencing is typically unable to discern intact from replication-defective integrated genomes and rarely able to predict response to therapy (48–50). A solution is the use of HIV-infectable reporter cell lines (e.g., MAGI, GHOST, or TZM-bl) that use luminometric or fluorometric methods (e.g., plate reader or flow cytometry) to confirm the presence of infectious virions and study replication. These methods can both enumerate viruses and be used to study strain-specific susceptibility to drugs or antibodies (51–55). However, current single-reporter systems provide limited information pertaining to genome variation and adaptations to the host.

Our aim in this study was to develop a cell-based strategy that exploits live cell imaging to not only detect HIV-1 infection but also mine single-cell data in a multivariate fashion, thus allowing us to access phenotypic information with enhanced data richness relative to prior approaches. To this end, we describe the creation and validation of the HIV-1 dual reporter sensor cell (DRSC) assay using cells designed to undergo multicolor visible transitions in response to infection, requiring the coordinated activities of three independent HIV-1 gene products (Tat, Rev, and Vif) to define an intact, fully-active genome. We validate our system by testing the DRSC assay’s capacity to survey the relative potency and functions of several subtypes and strain-specific *vif* alleles, and by modifying the assay to differentiate between X4-, R5-, and dual-tropic viruses. Taken together, the DRSC platform demonstrates the strength and potential of multi-color cell-imaging assays based on viral response sensors to detect and characterize HIV-1 viruses as well as to study strain-specific attributes of viral replication and host cell response.

## RESULTS

### Development of the dual reporter sensor cell assay

**Fig 1:**
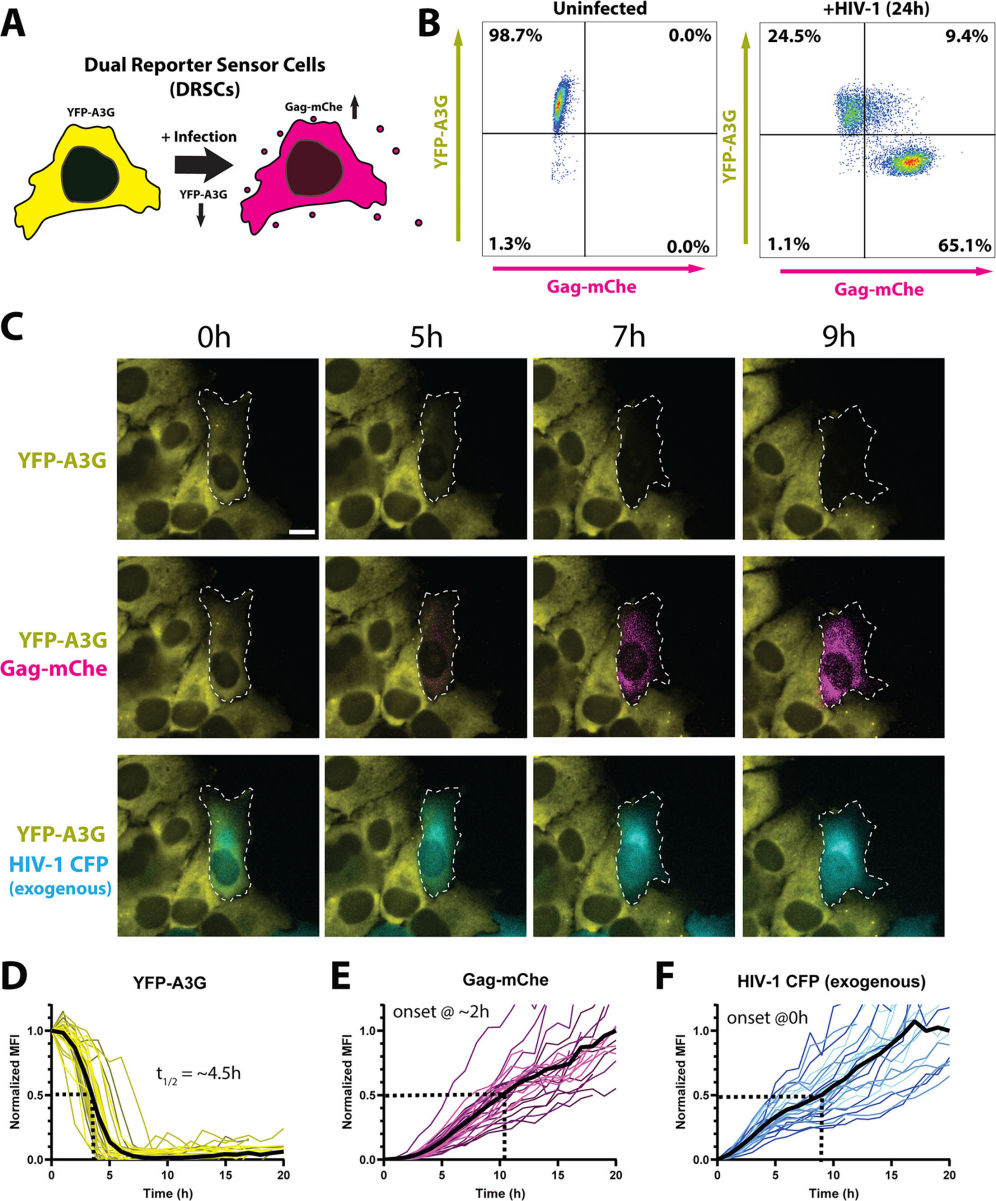
Dual reporter sensor cells (DRSCs). (**A**) Cartoon depiction of DRSCs and their color-switch transition from yellow (YFP-A3G(+)/Gag-mCherry(−)) to red (YFP-A3G(−)/Gag-mCherry(+)) after infection with HIV-1. (**B**) Flow cytometry analysis of YFP-A3G and Gag-mCherry fluorescence in uninfected DRSCs (left) and DRSCs infected with vesicular stomatitis virus G protein (VSV-G) pseudotyped HIV-1_NL4-3_-CFP reporter virus at a multiplicity of infection (MOI) of 1 (right) and fixed at 24 hours post-infection (hpi). (**C**) Individual images from live cell imaging of DRSC activation following infection with HIV-1_NL4-3_-CFP reporter virus (see also Movie S1). An infected cell undergoing a color switch is outlined in white. Scale bars represent 10 µm. (**D–F**) Single-cell analysis of DRSC YFP-A3G downregulation (Panel D), expression of the cell-intrinsic Gag-mCherry reporter (Panel E), and expression of the virally encoded CFP reporter (Panel F) following infection with VSV-G pseudotyped HIV-1_NL4-3_-CFP reporter virus at an MOI of 1. Mean fluorescence intensity (MFI) tracks from 30 cells from one of three representative experiments are shown. Average kinetics are indicated by the solid black line, with the dotted line indicating T_1/2_ for YFP-A3G decay and Max_1/2_ for Gag-mCherry and CFP expression normalized to average MFI at the 20 h time point. T = 0 represents the first detection of the exogenous viral CFP reporter.

To generate a multicolor cell-based assay capable of measuring multiple important virus traits in a single experiment, we engineered HeLa.CD4 DRSCs constitutively expressing a YFP-APOBEC3G (YFP-A3G) “OFF” reporter (Fig. 1A, yellow) and encoding a latent HIV-1 Gag-mCherry “ON” reporter (Fig. 1A, magenta). YFP-A3G was chosen as an “OFF” sensor because A3G is a host restriction factor against HIV-1 that is counteracted by the viral Vif protein, with viral infection and subsequent Vif expression leading to rapid ubiquitination and degradation of A3G through the proteasomal degradation pathway (56–64). Our “ON” sensor was an integrated subgenomic proviral reporter that expressed a fluorescent Gag-mCherry fusion protein activated by incoming viral expression of the viral Tat and Rev regulatory proteins; with Tat and Rev essential for efficient viral RNA transcription and late-stage RNA nuclear export, respectively (65, 66). Our ON reporter was similar in principle to several prior HIV-1 reporter systems that express fluorescent proteins in response to Tat expression (55, 67), but novel in that the readout was both Tat- and Rev-dependent and produced Gag-mCherry so that we could both measure late-stage viral gene activation and track Gag-mCherry in the context of its role in driving virus capsid assembly. Although HeLa.CD4 cells are derived from epithelial cells that are not native targets of HIV-1 infection *in vivo*, they were chosen for DRSCs because of their long-standing use in HIV replication studies and useful imaging qualities including flatness and their stationary nature amenable to long-term (>24 h) video microscopy.

Our prediction was that when HIV-1 infection occurred within a DRSC, viral Vif expression would eliminate the YFP-A3G reporter, while Tat and Rev expression would coordinately transactivate Gag-mCherry expression, resulting in an “ON/OFF” color-switch with loss of YFP-A3G and gain of Gag-mCherry [yielding a YFP(−)/mCherry(+) infected cell] (Fig. 1A). We tested this prediction by first analyzing DRSCs using flow cytometry after infecting the cells at a multiplicity of infection (MOI) of 1 with a subtype B-derived HIV-1_NL4-3_-CFP reporter virus (pseudotyped with the vesicular stomatitis virus G protein: VSV-G); observing that 65.1% of the cell population shifted from YFP(+)/mCherry(−) (uninfected) to YFP(−)/mCherry(+) at 24 hours post-infection (hpi), with an additional 9.36% of mCherry(+) DRSCs still YFP-A3G-positive, likely indicating that a subset of infected cells were still in the process of a color-switch (the infection was not synchronized) (Fig. 1B).

To define single-cell kinetics, DRSCs were infected using the same HIV-1_NL4-3_-CFP reporter virus prior to live cell imaging over a 20 h time course (Fig. 1C through F; Movie S1). Infected cells were tracked over time measuring changes to per cell YFP-A3G, Gag-mCherry, and viral CFP mean fluorescence intensities (MFIs). Consistent with the onset of Vif expression, YFP-A3G levels began to decline ~1.5 h after productive infection (defined as the onset of CFP expression) with a half-time (T_1/2_) of decay of 4.58 ± 1.27 h (*n* = 30), consistent with previous estimates of Vif-mediated A3G degradation kinetics (68–72) (Fig. 1C with quantification in Fig. 1D). Gag-mCherry expression was initiated ~2 h after the onset of CFP expression, consistent with the Gag-mCherry reporter representing a “late” (Rev-dependent) viral gene product encoded by unspliced viral RNA, in contrast to the “early” (Rev-independent) CFP reporter expressed from the *nef* locus, with both signals exhibiting linear rise kinetics (Fig. 1E and F) achieving half-maximum (1/2 max) times of ~9 h and ~10.5 h, respectively.

Taken together, DRSCs allowed for “color-switch” detection of HIV-1 infection and direct single-cell measurements of both viral Rev- and Tat-mediated activation of Gag-mCherry expression and Vif-mediated YFP-A3G degradation kinetics.

### Use of DRSCs to measure viral infectivity

**Fig 2:**
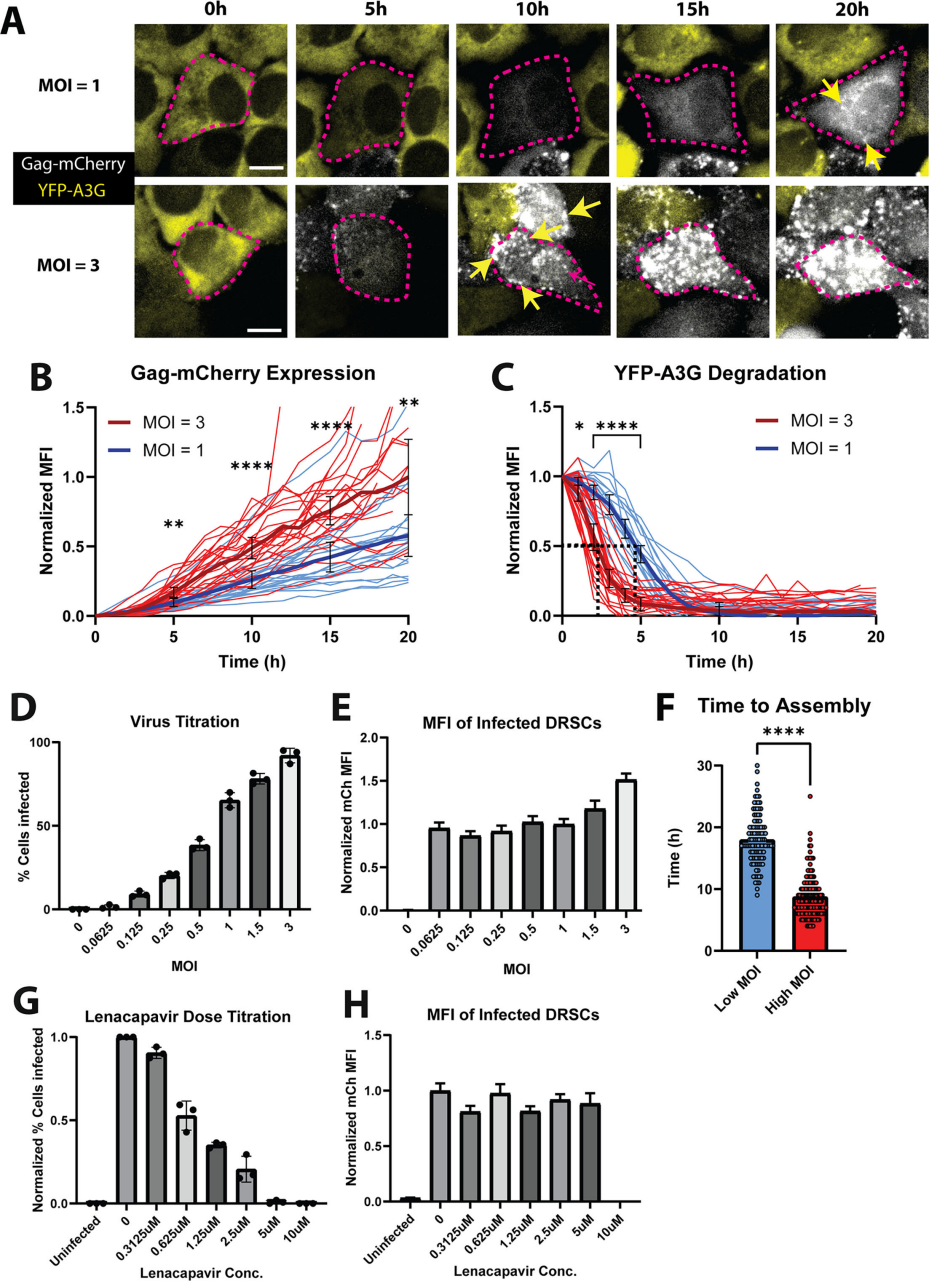
DRSCs for single-cell quantification of HIV-1 infection and virus particle assembly kinetics. (**A**) Individual images from live cell imaging of DRSC activation following infection with HIV-1_NL4-3_ virus (VSV-G pseudotyped) at an MOI of ~1 (low) or ~3 (high) (see also Movie S2). Scale bars represent 10 µm. YFP-A3G is shown in yellow and Gag-mCherry in white (for better visual clarity relative to magenta as in Fig. 1). (**B and C**) Quantification of DRSC Gag-mCherry reporter activation (**B**) and YFP-A3G reporter degradation kinetics (**C**) over time in a minimum of 20 individual cells from one of three representative live cell imaging time courses following infection at high (MOI = 3, red) or low (MOI = 1, blue) MOI. Averages for each condition are shown by thick lines. Error bars depict a 95% confidence interval. (**D**) Analysis of percent of DRSCs activated when infected with differing amounts of virus (HIV-1_NL4-3_ (E-R-/CFP)). Infected cells were counted in images taken for each condition in three independent infections. (**E**) Analysis of average Gag-mCh MFI of infected cells in each condition in (**D**) with the exception of the condition at MOI = 0, where the fluorescence in the mCh channel for uninfected cells is displayed. *N* = 45 cells. (**F**) Analysis of time to mCherry puncta formation by tracking Gag-mCherry fluorescence in DRSCs after initial infection through time-lapse live cell imaging. *N* > 100. (**G**) Analysis of percent of DRSCs activated when infected at an MOI of 1 (HIV-1_NL4-3_ (E-R-/CFP)) with differing amounts of Lenacapavir. Infected cells were counted in images taken for each condition in three independent infections. (**H**) Analysis of average Gag-mCh MFI of infected cells in each condition in (**G**). *N* = 45 except for infections treated with 10 µM (*N* = 0) or 5 µM (*N* = 4) Lenacapavir due to no or fewer cells infected under these conditions, respectively. Error bars depict ± SEM. *P*-values are depicted as follows: * <0.05, ** <0.01, **** <0.0001. Student’s t test was conducted to compare data for statistical significance.

We predicted that a useful feature of DRSCs would be their potential to not only detect infection but also measure differential levels of gene activation and MOI. To test this hypothesis, we compared the response of DRSCs to low (1) vs high (3) MOI infection, measuring single-cell responses in our live cell imaging assay (Fig. 2A; Movie S2). Consistent with increased MOI, we observed greater than twofold increases in Gag-mCherry levels over time for high MOI relative to the low MOI conditions (Fig. 2B). YFP-A3G degradation was also accelerated under the high MOI condition (T_1/2_ of ~2.5 h for MOI of 3 relative to T_1/2_ of ~4.5 h for MOI of 1) and initiated ~1 h earlier (Fig. 2C). These data illustrated the utility of the single-cell kinetic assay that allowed us to detect relatively modest differences to infection and gene activation levels.

To investigate the limits of viral detection, we conducted an additional experiment exploring the dynamic range of the assay through further fixed timepoint analysis (at 24 hpi) for a dose titration (Fig. 2D and E). Overall, we observed that dose titration yielded a linear decay in the number of infected cells, as expected. However, per cell MFI remained consistent at or below MOI = 1, consistent with our detecting a single integrated provirus in each infected cell at low MOI. In contrast, we detected higher per-cell MFI values at virus titers above MOI = 1, consistent with the detection of multiple integrated proviruses. Taken together, we concluded that we can interpret levels of Rev- and Tat-dependent activation of our Gag-mCherry reporter as a proxy measurement for the number of integrated proviruses.

We also predicted that the Gag-mCherry marker would allow us to monitor the extent of infection through a second method of tracking rates of virus particle assembly. Gag drives HIV-1 particle formation at the plasma membrane wherein ~1,500 Gag molecules multimerize to form the immature viral capsid that packages two copies of the viral RNA genome (73, 74). Particle assembly is driven through a cooperativity-based mechanism wherein Gag multimerization and subsequent membrane localization are triggered when the intracellular concentration of Gag reaches a threshold level (75–77). Because Gag multimerization is necessary for virion production, we hypothesized that higher MOI would lead to faster kinetics of virion assembly as defined by Gag-mCherry reporters forming bright puncta at the cell’s plasma membrane. As expected, we observed a more rapid appearance of Gag-mCherry puncta for high MOI infection (8.76 hpi ± 3.43 h) relative to low MOI infection (18.02 hpi ± 4.08 h) (Fig. 2A and F), illustrating that DRSCs can report on levels of viral gene activity based on virion production kinetics.

Reporter cells are used extensively in HIV-1 research to test antiviral inhibitors, so we tested the ability of DRSCs to measure HIV-1 inhibition by the capsid inhibitor Lenacapavir (78). To this end, DRSCs were infected with VSV-G pseudotyped HIV-1_NL4-3_ at an MOI of 1, with cells analyzed at 24 hpi for the percent of cells infected (Fig. 2G) as well as the per-cell Gag-mCherry MFI (Fig. 2H). As expected, we detected dose-dependent inhibition of infectivity, with Lenacapavir reducing the number of cells infected but not affecting per-cell Gag-mCherry expression levels, consistent with the drug acting to prevent proviral integration but not affecting viral gene expression.

### DRSCs can be used to delineate strain-specific HIV-1 Vif activities

**Fig 3:**
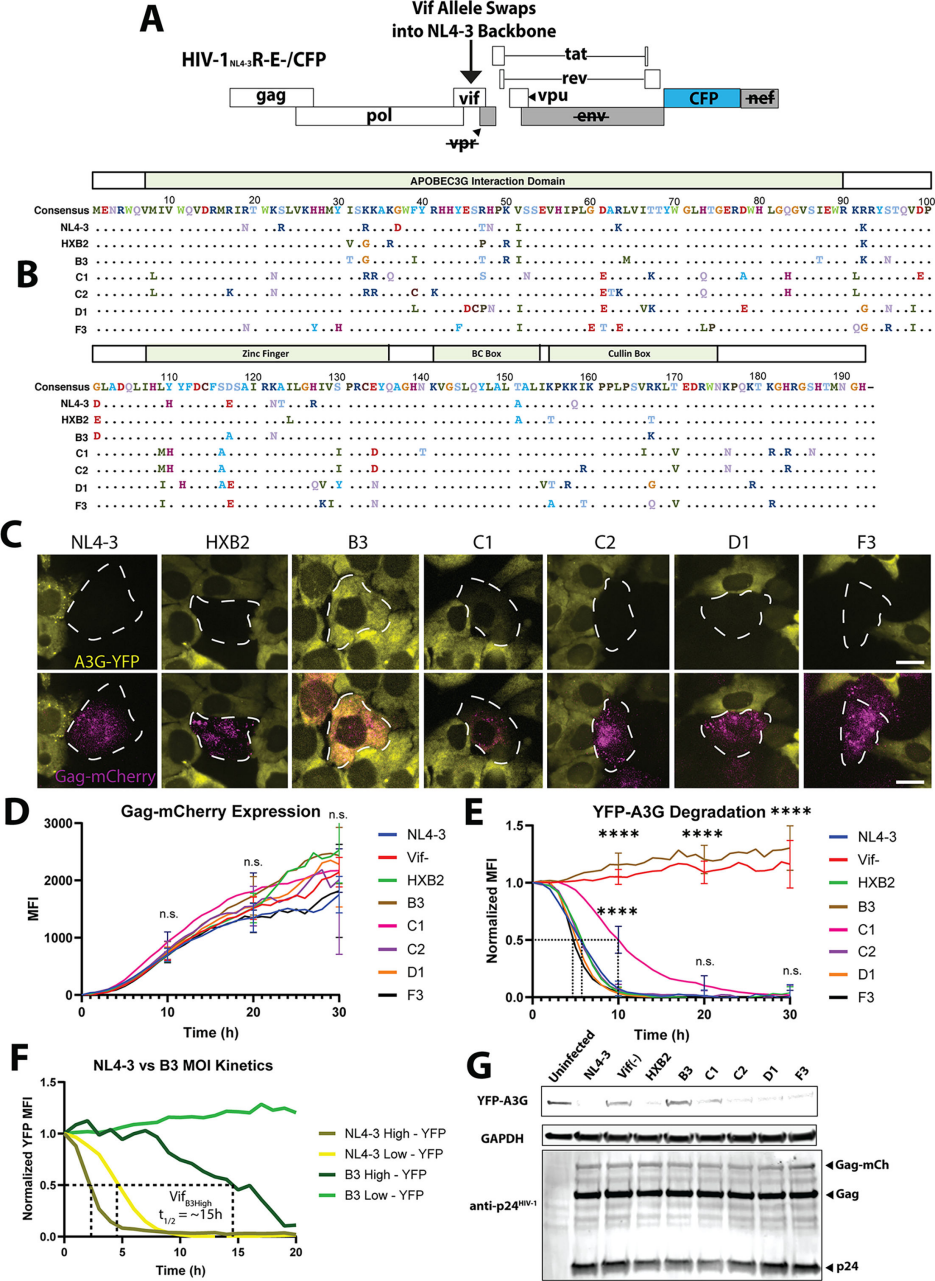
YFP-A3G degradation kinetics for vif alleles derived from patient isolates. (**A**) Depiction of chimeric HIV-1_NL4-3_ (E-R-/CFP) virus genome backbone into which *vif* alleles were swapped. (**B**) Depiction of *vif* alleles used in this experiment and their amino acid differences. (**C**) Images of YFP-A3G and Gag-mCherry fluorescence in DRSCs infected with the panel of *vif*-swapped chimeric viruses at 10 hpi. Scale bars represent 10 µm. (**D**) Quantification of Gag-mCherry expression over time in DRSCs infected with the chimeric *vif*-swapped viruses at an MOI of 1. (**E**) Quantification of YFP-A3G downregulation over time in DRSCs infected with the chimeric *vif*-swapped viruses at an MOI of 1 from a live cell imaging time course with individual cell fluorescence tracked for 30 h for each *vif* allele (see also Movie S3). *N* ≥ 30 cells tracked for each *vif* allele infection from one representative experiment. Average kinetics for each *vif* allele are shown. (**F**) Quantification of B3 Vif infection YFP-A3G degradation kinetics under low (MOI = 1) and high (MOI = 3) MOI conditions and compared with NL4-3 Vif high and low MOI infection (see also Movie S3). (**G**) Western blot analysis of DRSCs infected with the panel of *vif*-swapped chimeric viruses at 36 hpi (MOI = 1). Blots were incubated with anti-A3G and anti-p24^HIV-1^. Error bars depict a 95% confidence interval. One-way analysis of variance (ANOVA) was used for statistical comparison of *vif* allele fluorescence kinetics to NL4-3 Vif infection data. *P*-values are depicted as follows: **** <0.0001. n.s., not significant.

HIV-1 exhibits one of the highest known mutation rates of all viruses (79, 80). Large sources of genetic variability occur within the HIV-1 regulatory and accessory genes *tat*, *rev*, *vif*, and *vpr*, and several studies have implicated this variability as of important relevance to pathogenic outcomes in humans (30–45). To test if the DRSC platform could be used for detection of accessory protein heterogeneity in this context, we engineered HIV-1_NL4-3_ reporter viruses wherein we replaced the native *vif* allele with alleles derived from a diverse panel of clinical strains, previously demonstrated by Binka et al. to be associated with differences to A3G-degradation capabilities and overall viral replication fitness (39) (Fig. 3A). The amino acid similarity of the *vif* alleles tested is shown in Fig. 3B, with the *vif* alleles having an average difference of 9.1% (17.5 AA) when compared to NL4-3, and with C1 being the most divergent [with a 12.4% (24 AA) variance]. The Vif residues implicated in A3G binding and degradation by Vif are present in the N-terminus and C-terminus, respectively (81–83). Additional controls included a virus wherein we inactivated native *vif_NL4_*_-3_ using mutagenesis (Vif(−) = K26stop, H27stop) or replaced the *vif_NL4_*_-3_ allele with *vif* from an alternative lab strain (HXB2) known to degrade A3G with similar potency (39, 84–86).

We utilized single-cell tracking of DRSCs infected with each virus at an MOI of 1 to track rates of YFP-A3G degradation for each *vif* allele expressed in an otherwise identical virus genome backbone. Based on the Gag-mCherry response rates, none of the *vif* alleles notably affected Tat/Rev driven gene expression over a 30 h time course (Fig. 3C and D). Relative to Vif_NL4-3_, similar YFP-A3G degradation rates were observed for each *vif* allele tested with the exception of B3 and C1 (Fig. 3C and E; Movie S3), with the B3 and C1 results consistent with decreased potency measurements previously reported in Binka et al. (39). C1 Vif degraded YFP-A3G but at a significantly slower rate (T_1/2_ of ~10 h) relative to Vif_NL4-3_. By contrast, B3 Vif was only able to degrade YFP-A3G when cells were infected at an elevated MOI, and even then with relatively slow kinetics (T_1/2_ = ~15 h) (Fig. 3F). Interestingly, when infecting with B3 Vif virus at high MOI, live-cell imaging revealed YFP-A3G localized to Gag-mCherry puncta during infection, consistent with A3G being removed from the cytoplasm by B3 Vif too slowly to prevent A3G incorporation into virions (Movie S3). We confirmed the relative A3G degradation capabilities of our panel of *vif* alleles by conducting western blot analysis on lysates of DRSCs infected with the *vif*-swapped viruses (Fig. 3G). These results validated DRSCs as a sensitive tool capable of detecting variability within the Vif function and achieving the granularity needed to reveal differences in the net capacity of Vif to degrade and suppress YFP-A3G over the duration of the infectious cycle.

### DRSCs for unbiased measurements of host cell cycle dysregulation

**Fig 4:**
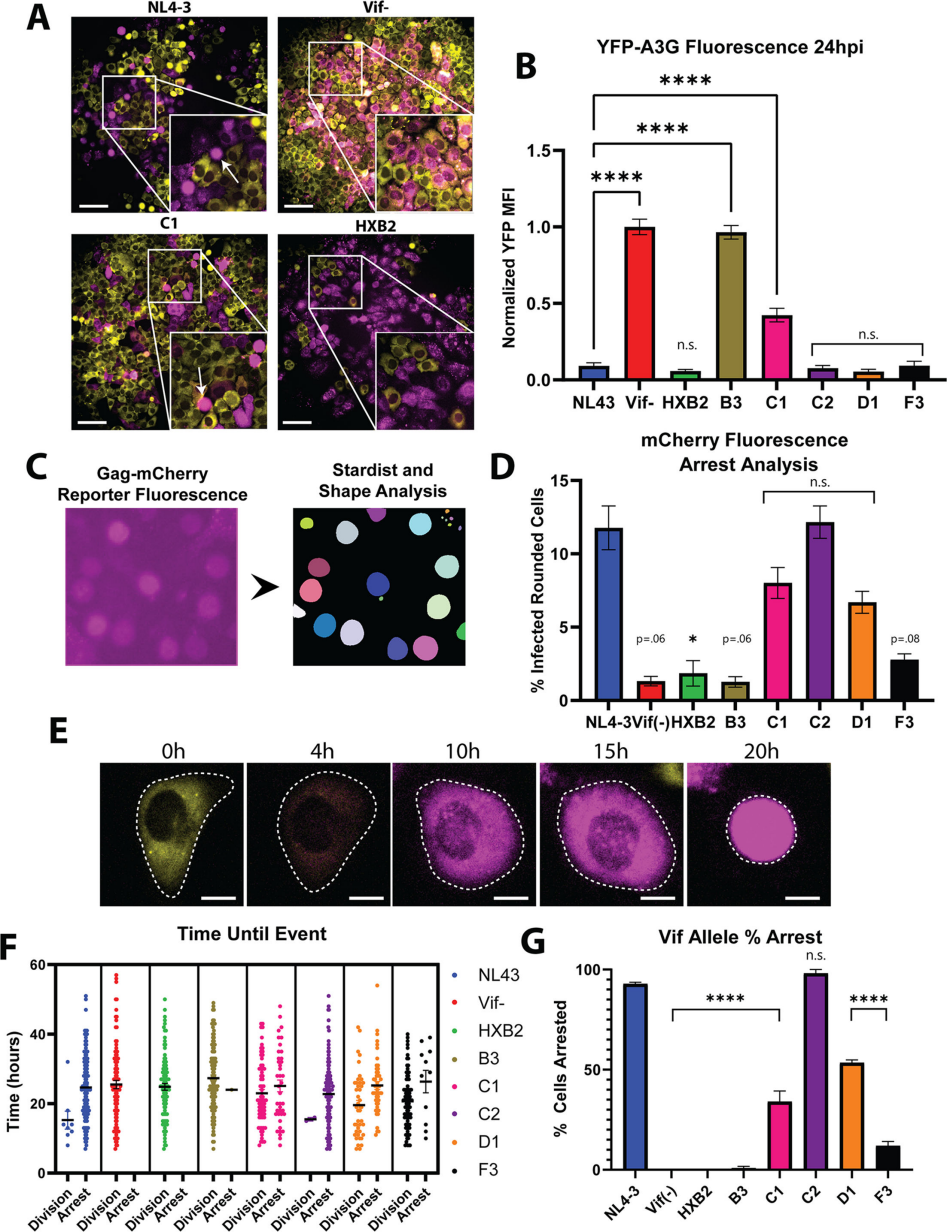
Vif-mediated cell cycle arrest varies by vif allele. (**A**) Images of DRSCs infected with NL4-3, Vif(−), C1, and HXB2 Vif chimeric viruses 48 hpi, with examples of cells undergoing arrest and rounding up pointed to by arrows. Scale bars represent 100 µm. (**B**) Quantification of YFP-A3G levels within single DRSCs infected with chimeric *vif* allele viruses (24 hpi) through Cellpose segmentation. *N* = 3. (**C**) Example of DRSC mCherry reporter fluorescence being segmented into masks through Stardist analysis. (**D**) Quantification of % of rounded cells from all infected cells per image of DRSCs infected with our panel of chimeric *vif*-swapped viruses using a FIJI-Stardist workflow. A minimum of 100 infected cells in each of three images of infected cells 48 hpi from each of our *vif* allele movies were analyzed. (**E**) Images of a DRSC depicting fluorescence and morphology changes from initial infection (with HIV-1_NL4-3_ (E-R-/CFP) virus) of a DRSC to cell cycle arrest (rounding up). Scale bars represent 10 µm. (**F**) Manual quantification of time to arrest or division after onset of infection for DRSCs infected with our panel of *vif*-swapped viruses and tracked through live cell imaging. *N* ≥ 100 cells tracked for each *vif* allele infection. (**G**) Quantification of % of cells that underwent cell cycle arrest from data in F. Infections were conducted at an MOI of 1. Graphs depict ± SEM. One-way ANOVA was used for statistical comparison to NL4-3 Vif. Only significant differences are shown. *P*-values are depicted as follows: * <0.05, **** <0.0001.

In Fig. 3 we established that single-cell tracking can reveal differential Vif-mediated A3G degradation capabilities and kinetics. Because live-cell imaging is not available in all settings, our next goal was to assess the capacity of the DRSC platform to detect viral heterogeneity in a fixed cell population at a single timepoint post-infection (Fig. 4). To this end, we used Cellpose, a deep learning-based segmentation software (87), to detect and segment individual infected cells expressing the Gag-mCherry “ON” signal prior to measuring single-cell YFP-A3G levels. Fig. 4A compares NL4-3 (WT), Vif(−), C1, and HXB2 chimeric Vif infection conditions at 24 hpi. Consistent with live cell imaging, our Cellpose single timepoint analysis in Fig. 4B demonstrated that WT-infected cells displayed low levels of YFP-A3G fluorescence relative to Vif(−) infected cells, similar to most other *vif* allele infections, with C1 Vif exhibiting an intermediate phenotype and B3 Vif showing similar intracellular levels of YFP-A3G as the Vif(−) infection. NL4-3, Vif(−), and C1 infected cells exhibited average normalized YFP-A3G levels of 0.15, 1.0, and 0.36, respectively, consistent with C1 Vif exhibiting a slower relative rate of A3G degradation.

A second useful attribute of machine learning-based image analysis tools is their capacity to measure cell morphological changes that might relate to HIV-1 pathogenicity. For example, in addition to degrading A3G, Vif was recently shown to induce cell cycle arrest through antagonism of host PP2A regulatory proteins (88–90). PP2A phosphatase regulator PPP2R5 subunits (A–E) have been shown to be degraded by some, but not all, patient-derived and laboratory Vif isolates. Importantly, the two lab strains included in our analysis (NL4-3 and HXB2) are known to exhibit different phenotypes, wherein Vif_NL4-3_ induces arrest, while Vif_HXB2_ does not (69, 86, 91). Accordingly, we next asked if DRSCs could be used to track cell morphology changes consistent with Vif’s effects on the cell cycle. To this end, we generated an image analysis workflow to measure cell morphology differences in an unbiased manner using the FIJI plug-in Stardist (https://imagej.net/plugins/stardist) (92) (Fig. 4C), with this analysis demonstrating marked variability in cell morphology for several of the *vif* alleles tested. NL4-3 and C2 Vifs caused the highest degree of change (cell rounding consistent with cell cycle arrest) as we have previously described (71, 93), while B3, F3, and HXB2 Vif had little to no effect on cell morphology, more closely matching the Vif(−) control (Fig. 4D). C1 and D1 Vifs caused intermediate levels of arrest when compared to C2 and NL4-3.

To validate the unbiased, automated “single-image” approach, we re-analyzed our *vif* allele infection time-lapse movies (see Fig. 3), tracking individual HIV(+) cells to determine whether they underwent cell cycle arrest or accomplished cell division (Fig. 4E). These measurements closely matched the results from our automated approach (Fig. 4F and G) but with the kinetic analysis providing more data granularity including the degree and timing of arrest. The C2 allele caused arrest levels similar to NL4-3 (98.1% and 92.9%, respectively), with B3 and F3 Vifs rarely causing arrest (0.8% and 11.9%, respectively), and C1 and D1 Vif infections causing an intermediate phenotype (arrest in 34% and 53.4% of infected cells, respectively). These results were consistent with a recent report from Salamango et al. that demonstrated NL4-3 Vif to be a strong degrader of PPP2R5 while several patient isolate Vifs were less active in this function (69, 94).

In summary, in addition to differences to Vif allele-specific effects on YFP-A3G degradation, DRSCs can also readily allow for detection of differential viral effects on cell morphology through single-timepoint analysis, in this case impacting the cell cycle.

### Use of DRSCs to differentiate between R5- and X4-tropic viruses

HIV-1 has a main cell receptor, CD4, but uses either of two coreceptors, CXCR4 (X4) or CCR5 (R5) (95–101). Most transmissible strains of HIV are R5-tropic, with the virus sometimes developing X4-tropism during late-stage infection, and with some strains being dual-tropic (X4R5) (102–104). Some HIV-1 subtypes have been found to cause higher mortality rates than others, particularly subtype D, and are attributable, at least in part, to dual-tropism (8, 10, 13, 14, 83–86). Moreover, defining co-receptor usage is necessary for patients undergoing combination antiretroviral drug treatment with a co-receptor inhibitor (105). Predictions of receptor tropism can be made by sequencing the *env* region of the genome, but such predictions can be unreliable (106–108).

**Fig 5:**
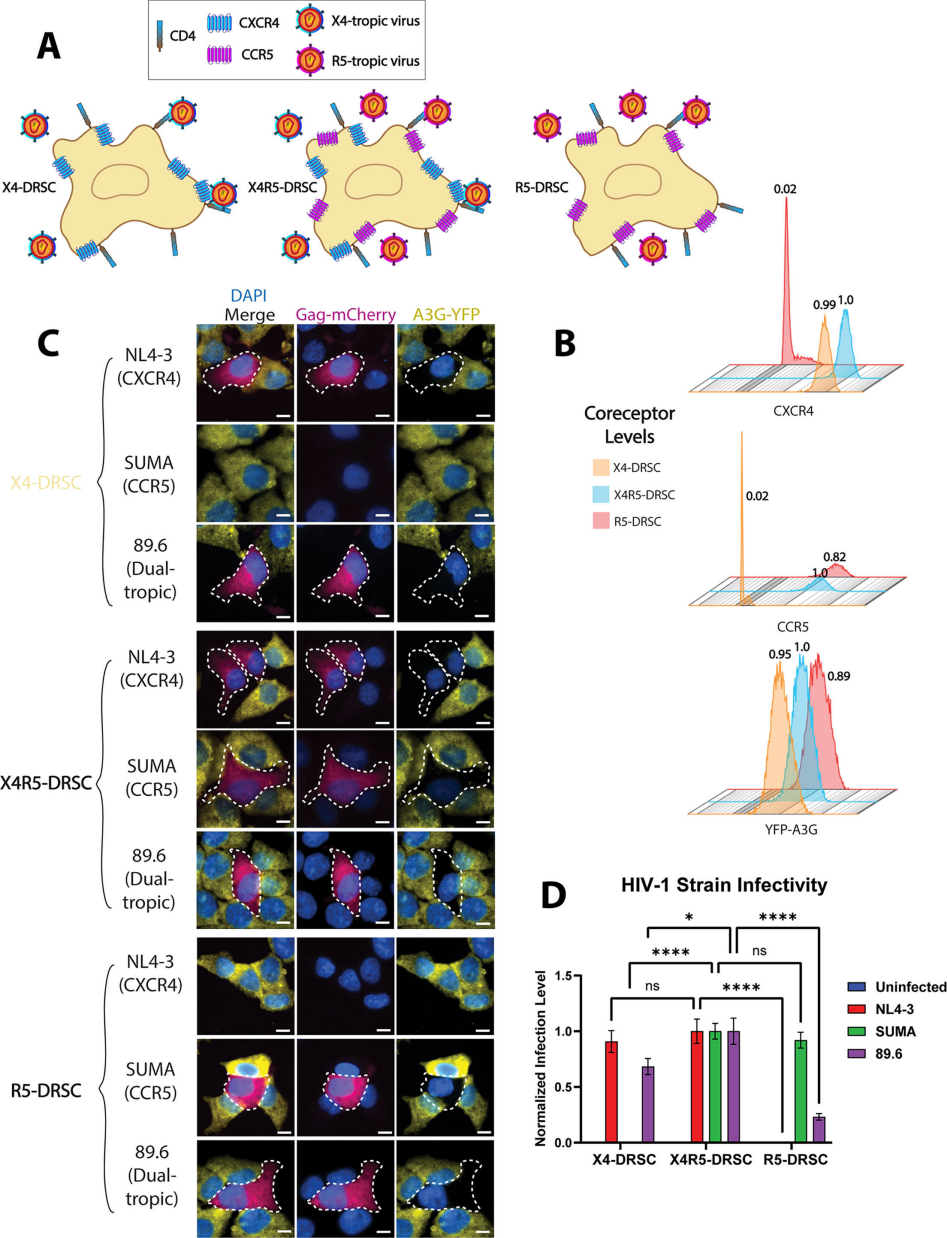
DRSC panel for HIV-1 coreceptor tropism. (**A**) Cartoon depiction of DRSC coreceptor panel. (**B**) Flow cytometry histograms depicting CXCR4, CCR5, and YFP-A3G levels for X4-, X4R5-, and R5-DRSCs. Mean coreceptor and YFP levels relative to X4R5-DRSCs are displayed above each corresponding histogram. (**C**) Images of X4-, X4R5-, and R5-DRSCs infected with unattenuated X4-tropic NL4-3, R5-tropic SUMA, and dual-tropic 89.6 HIV-1 viruses at an MOI of 1. Cells were fixed at 24 hpi. Scale bars represent 10 µm. (**D**) Quantification of the number of DRSCs infected in three separate infections with each virus (NL4-3, SUMA, 89.6) per 20× image. Titer was performed on X4R5-DRSCs, and cells were infected with a virus that caused infection at an MOI = 1 in X4R5-DRSCs. Number of infected cells in each image was normalized to the average number of infected cells for each virus in X4R5-DRSCs. One-way ANOVA was used for statistical comparison. *P*-values are depicted as follows: * <0.05, **** <0.0001.

To differentiate between X4 and R5 viruses, we engineered DRSCs that expressed either CXCR4 or CCR5, or both (Fig. 5A) and confirmed co-receptor expression using flow cytometry (Fig. 5B). To test if this coreceptor panel of DRSCs would then allow for determination of coreceptor tropism of incoming virus by detecting the presence of infected YFP-A3G(−)/Gag-mCherry(+) cells, our panel of DRSCs was subsequently infected with HIV-1_NL4-3_ (X4-tropic), HIV-1_SUMA_ (R5-tropic transmitted founder isolate), or HIV-1_89.6_ (dual-tropic transmitted founder isolate). As expected, the X4-tropic NL4-3 virus infected both X4- and X4R5-DRSCs, the R5-tropic SUMA virus infected the R5- and X4R5-DRSCs, and the dual-tropic 89.6 infected all DRSC types (Fig. 5C and D). Interestingly, we found that the dual-tropic 89.6 virus caused higher levels of infection in the X4- and X4R5-DRSCs than in our R5-DRSCs, potentially indicating that the 89.6 virus infection was more efficient in utilizing the CXCR4 coreceptor relative to CCR5 (Fig. 5D). In conclusion, in addition to detecting virus and measuring virological and cell responses, a panel of coreceptor-specific DRSCs can also be used to define the relative receptor tropism of HIV-1 viruses.

## DISCUSSION

Here, we describe the creation of the DRSC assay as a proof-of-concept cell system for detecting, quantifying, and carrying out multivariate analysis of HIV-1 replication. In this system, HeLa-CD4 cells were engineered to express two independent HIV infection-linked reporters; a degradation-based “OFF” YFP-tagged biosensor and a virus-responsive “ON” mCherry-tagged biosensor. We demonstrated using these “ON-OFF” markers that we can both detect HIV-1 and elucidate single-cell attributes of virus-host interactions of potential relevance to disease progression. First, the YFP-A3G and Gag-mCherry reporters allowed us to measure the level of infection by directly monitoring the kinetics of YFP-A3G degradation coupled to Gag-mCherry activation and virus particle assembly (Fig. 1 and 2). Second, comparisons of viruses encoding strain-specific *vif* alleles demonstrated heterogeneity in the relative capacities of differing Vif proteins to downregulate A3G and cause cell cycle effects (Fig. 3 and 4). Third, generating distinct versions of DRSCs that express either the CCR5 or CXCR4 co-receptor provided even more granularity, allowing us to distinguish between R5-, X4-, and dual-tropic viruses and confirming that DRSCs can be activated by transmitted/founder strains of HIV-1.

Taken together, our DRSC platform provides an example of how cell-based systems and multicolor imaging can be applied for multivariate analyses and detection of intact HIV-1 viruses. These tools and approaches are intended to be useful for basic research studies of HIV-host interactions, screening for new therapies, and detecting and characterizing patient-derived viruses.

Cell-based reporters have traditionally played major roles in detecting and quantifying HIV-1 infection in diverse contexts including virus titering, assaying for antibody neutralization (109), studying inhibition by pharmaceutical drugs or antibodies (53), and tracking the latent patient reservoir (53, 110). However, to date most cell-based modalities for detecting replication-competent HIV-1 and studying viral dynamics are one-dimensional; attractive in their simplicity but leaving large amounts of untapped data. We recognize that a potential limitation of the assay is that DRSCs are not a T-cell-derived reporter cell line. However, HeLa cell-based reporters (e.g., TZM-bl) have been used extensively for HIV-1 studies including for the detection of patient isolate viruses (54, 111, 112) and, as previously noted, are far superior to T cells (or monocyte-derived cell lines) for tracking cellular and viral trafficking and measuring single-cell dynamics due to their stationary nature and spread morphology exhibiting a relatively large nucleus and abundance of cytoplasm.

The popular TZM-bl and GHOST reporter systems are Tat-responsive (LTR-driven) and thus only require a single HIV-1 gene to be functional within the integrated genome to produce a single positive signal. Sup-GGR, a recently described dual-reporter cell line for HIV-1, has improvements in that it expresses both Gaussia luciferase and hrGFP in a Tat- and Rev-dependent manner, thus requiring the presence of functional versions of both viral auxiliary proteins (113). Our DRSC strategy is novel in that it combines two functionally relevant visible reporters (Gag-mCherry and YFP-A3G) with the reporters used to partially “genotype” the infecting virus using open-source image analysis pipelines. Antiviral resistance prediction has historically relied largely on sequencing to determine whether a patient’s virus is susceptible to a given antiviral. Since newer antiviral therapies are being designed to have higher genetic barriers to resistance, more complex and strain-specific resistance patterns are bound to emerge that are more difficult to detect via sequencing. Therefore, phenotypic assays like the described DRSC assay could be particularly useful for patients who have failed multiple regimens (114). We do note, however, that our assay is by its nature limited to reporters detecting what they can detect so although DRSCs could have an advantage over sequencing in that they will only detect intact, replication-competent genomes, the cells would only report on defects to Tat, Rev, or Vif function and may miss mutations in other parts of the genome. However, coupling DRSC infection to genome sequencing could serve as a means to generate a more complete picture of any patient-derived viral quasispecies.

**Fig 6:**
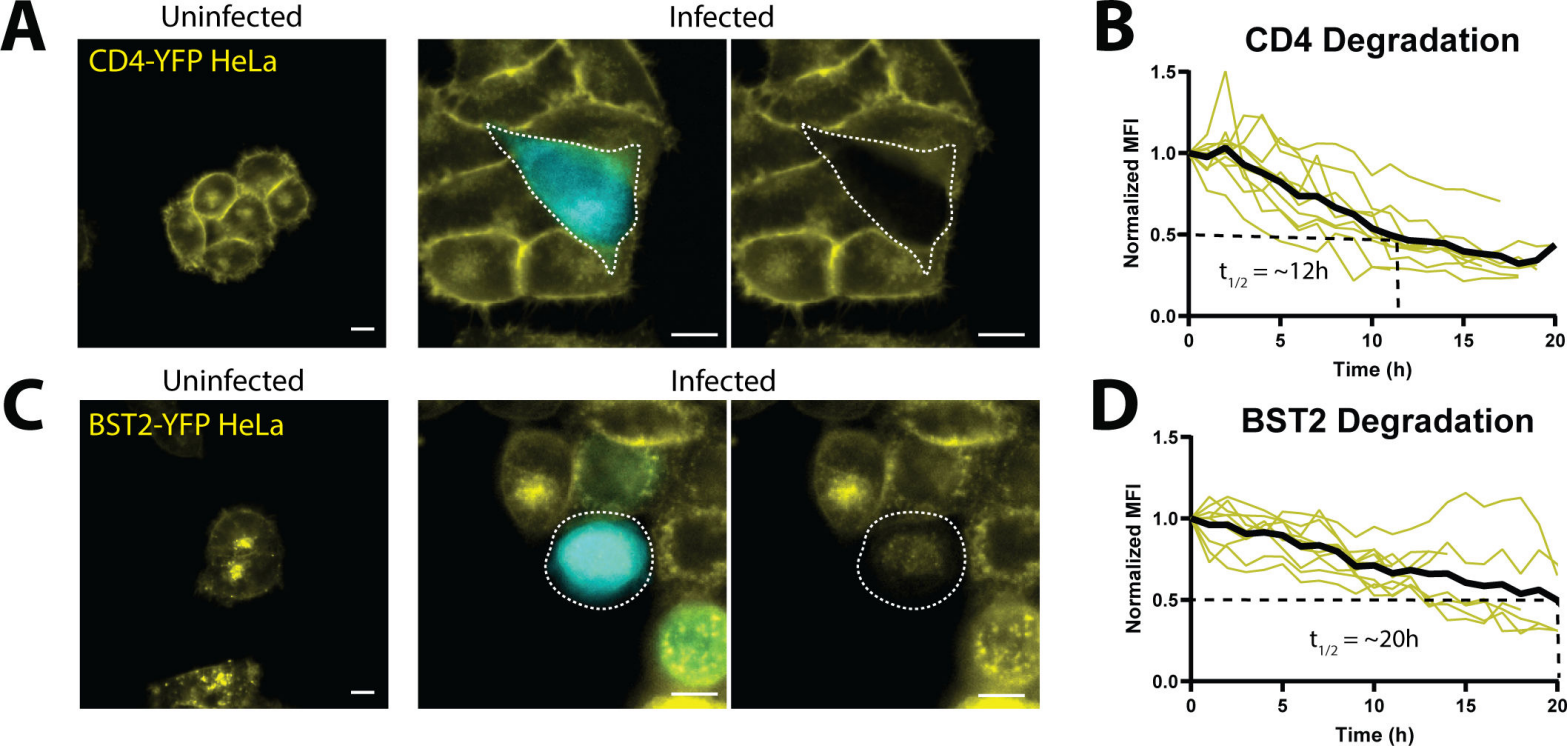
HIV-1 mediated degradation of YFP-tagged host factors. (**A**) Example of uninfected YFP-tagged CD4 reporter cells and cells 20 h after infection with an NL4-3/CFP reporter virus at an MOI of 1. (**B**) Kinetic analysis of CD4-YFP degradation by HIV-1 in 10 infected CD4-YFP reporter cells subjected to live cell imaging after infection at an MOI of 1. (**C**) Example of uninfected YFP-tagged BST2 reporter cells and cells 20 h after infection with an NL4-3/CFP reporter virus. (**D**) Kinetic analysis of BST2-YFP degradation by HIV-1 in 10 infected BST2-YFP reporter cells subjected to live cell imaging after infection. Individual cell kinetics are depicted by yellow lines, the average of the 10 cells analyzed per reporter cell infection is represented by the solid black line, and T_1/2_ is depicted by the dotted black line. T = 0 was treated as the onset of CFP expression after infection. Scale bars represent 10 µm.

DRSCs could also, in principle, be used in the context of high-content drug screens as a multivariate approach reporting on several key targets simultaneously, e.g., gene activation (Tat/Rev activity), Vif function, and Gag multimerization for virion assembly inhibitors. In this context, we note that the use of additional loss-of-signal fluorescent reporters linked to alternative host response proteins will provide future avenues for DRSC development. For example, we have already engineered versions of DRSCs that express reporters linked to BST2 (also known as Tetherin), CD4, or SERINC5 that can be used to view the differential host protein degradation activities of *vpu*, *env*, or *nef* alleles from different subtypes of HIV-1 (Fig. 6) (115–121). Although the kinetics of degradation are slower for these reporters relative to A3G, they illustrate the potential power of including additional reporters attached to functional proteins in a DRSC format, allowing for even richer multivariate screens of viral activities in a high-throughput format.

Our finding that *vif* alleles can exhibit different A3G degradation kinetics and cause markedly different effects on the cell cycle arrest further demonstrates the benefits of using single-cell dynamics-based assays to study virus–host interactions. Accessing these combined traits would not be feasible using traditional low-content biochemical read-outs. Variability in Vif function may be related to pathogenesis, as specifically in the case of A3G degradation, it has been reported that a suboptimal degradation of A3G may be beneficial to the virus in promoting progeny genome variation, developing antiretroviral inhibitor resistance, and possibly leading to faster progression of pathogenesis (37–40, 122–125). Thus, Vifs that may be “defective” but still functional in A3G degradation such as the C1 *vif* allele we tested may indeed play a large part in HIV-1’s evasion of treatment efforts. Why B3 Vif acted as a null mutant in regard to both A3G degradation and cell cycle arrest is unknown. Binka et al. previously reported B3 Vif as being expressed at equal levels compared to other Vifs tested (39), but exhibited loss-of-function, suggesting to us that the defect maps to the L64M and I87T mutations, of which only I87 has been implicated in Vif-mediated A3G degradation (126).

The detected heterogeneous abilities of the different *vif* alleles we tested to cause cell cycle arrest may also be relevant to the pathogenicity of HIV-1 strains. PP2A enzymes form complexes with PPP2R5 subunits which control substrate identification and are necessary for the function of the complex (127–129). PPP2R5 subunits are important for the replication of other viruses and their degradation is highly conserved among viral Vifs, including HIV-1 Vif (69, 130–132). Their degradation may be beneficial to HIV-1 as PPP2R5 may contribute to immune activation and control of protein translation (132, 133). Our arrest analysis results matched those previously reported for NL4-3 Vif having a high ability to induce cell cycle arrest, which consequently has been shown to be due to PPP2R5 subunit degradation.

In summary, the DRSC platform provides a novel framework for applying single-cell imaging to identify functional viral genomes and elucidate various viral attributes from both basic HIV-1 studies and in determining patient viral phenotype, potentially relevant to disease progression.

## MATERIALS AND METHODS

### Cell culture

Human HeLa cervical cancer cells and human embryonic kidney 293T (HEK293T) cells were purchased from the American Type Culture Collection. Cells were maintained at 37°C and 5% CO_2_ and cultured in Dulbecco’s Modified Eagle Medium (Gibco, cat. 11965092) supplemented with 10% heat-inactivated fetal bovine serum (FBS, Sigma, cat. F2442) and 1% penicillin-streptomycin-L-glutamine solution (PSG, Sigma, cat. G6784).

### Plasmids and virus production

Our CFP-expressing HIV-1 proviral reporter viruses were originally derived from an established NL4-3 molecular clone (pNL4-3/E-R-/luc, a kind gift of Dr. Nathaniel Landau and obtained through the NIH HIV Reagent Program) (134) that expressed firefly luciferase from the *nef* locus and contained inactivating point mutations within the *env* and *vpr* reading frames for enhanced biosafety. In our versions (pNL4-3/E-R-/CFP), the luciferase reporter was replaced with CFP to allow for fluorescence-based detection of infected cells (135). The *vif* alleles used for the analyses presented in Fig. 3 and 4 were a generous gift of Dr. Viviana Simon (Mt. Sinai School of Medicine, New York, NY) (39). *Vif* allele swaps were conducted through the use of overlapping PCR to insert desired *vif* alleles into our pNL4-3/E-R-/CFP backbone using PacI and AgeI cut sites. To generate a reporter virus, HEK293T cells were transfected with the pNL4-3/E-R-/CFP plasmids along with VSV-G and polyethylenimine. Media was exchanged 6 h post-transfection. VSV-G pseudotyped virus-like particles (VLPs) were then collected 48 h post-transfection from HEK293T cell media by filtering spent media through a 0.45 µm syringe filter and were frozen at −80°C until use. Viral stocks were titered using DRSCs.

For co-receptor studies (Fig. 5), full-length NL4-3, SUMA, and 89.6 viruses were generated from proviral plasmids transfected into HEK293T cells as described above. Infectious molecular clones for these viruses were obtained through the NIH HIV Reagent Program as kind gifts of Drs. M. Martin, John Kappes, Christina Ochsenbauer, and Ronald G. Collman (136, 137).

### Creation of DRSCs

DRSCs were engineered through retroviral transduction of *YFP-A3G* and HIV-1-responsive *Gag-mCherry* transgenes. The retroviral vector for YFP-hA3G (derived from pYFP-hA3G, a generous gift of Dr. Michael Malim, King’s College London) has been described previously (71). The HIV-responsive Gag-mCherry reporter was engineered as a derivative of plasmid pGPV-RRE (138) encoding the first half of the HIV-1 genome encoding *gag-pol* and *vif* upstream of a Rev Response Element (RRE) followed by the HIV-1 3’ long-terminal repeat region. To generate the Gag-mCherry fusion and abrogate Gag-Pol expression, the mCherry coding region was inserted into SacII and BsmB1 sites downstream of the Gag coding region (139, 140), yielding a C-terminal Gag-mCherry fusion protein. So that we could detect incoming Vif, the reporter’s *vif* allele was inactivated by inserting stop codons at the lysine-26 and histidine-27 codons. The vector also retained a small portion of the *vpr* coding region (encoding the first 17 amino acids) but lacked all other viral genes including *Vpu, Env, Rev, Tat, or Nef*. To generate a single-round vector, the *Gag-mCherry* plasmid (pNS341) was co-expressed with an HIV-1 packaging plasmid (psPax2) and VSV-G (141) to generate a “mini-virus” that was subsequently used to transduce YFP-A3G target cells. Single-cell DRSC clones were then isolated and screened for good response kinetics. CD4-YFP and YFP-BST2 DRSCs were generated similarly to YFP-A3G DRSCs, using either a previously described CD4-YFP retrovector (140, 142) or a new YFP-BST2 retrovector encoding the *yfp* reading frame fused to the *BST2* reading frame to generate an N-terminal YFP-tag separated from BST2 by a glycine-rich linker.

CD4 and CCR5 receptors were then added to DRSCs through retroviral transduction, with single clones with positive receptor expression selected for expansion. To create CXCR4 knock-out DRSCs, we utilized lentiviral-mediated CRISPR Cas9. lentiCRISPR v2 was a gift from Dr. Feng Zhang (Addgene plasmid # 52961; https://www.addgene.org/52961/) (143). gRNAs for CRISPR targets of the human CXCR4 gene were designed using the IDT gRNA design tool and were inserted into the lentiCRISPRv2 plasmid according to the Zhang Lab Lentiviral CRISPR Toolbox protocol. All lentiviral transductions were conducted with VSV-G pseudotyped virus with spinoculation for 2 h at 2,000 × *g*. The resulting cells were then single-cell cloned after sorting through flow cytometry and screened for the presence of proper receptors/reporters.

### Microscopy and image analysis

Cells for live cell imaging were plated at 40% confluency in 8-well glass-bottom plates (Ibidi, cat. 80827) and infected by adding VLP containing supernatant and incubating for 8 h before the start of live-cell imaging. For low MOI movies, 50 uL of virus-containing media supplemented was added to each Ibidi well, and 150 uL of VLPs were added to each well for high MOI conditions. All movies and images were acquired using 20× or 40× objective lenses on either a Nikon Ti-Eclipse inverted wide-field microscope using an Orca-Flash4.0 C11440 digital complementary metal oxide semiconductor camera or a Nikon A1R confocal microscope equipped with a Pathology Devices Live Cell stage-top incubation system which maintained cells at 37°C, 5% CO_2_, and 50% humidity. In most experiments, multichannel images were acquired every 60 min for 72 to 96 h, starting at 8 hpi.

For live cell analyses, randomly selected cells from three independent acquisition points were analyzed for each condition by manually measuring the MFI at each 60 min time point image of each corresponding fluorescent signal within the cell of interest from the start of infection (1 h before CFP expression onset) until the end of the movie or when the cell underwent cell cycle arrest or division. Cell fate analysis was conducted by tracking a minimum of 100 cells from each *vif* allele infection and measuring the time from onset of infection (1 h before CFP expression) until cell division or arrest. For fixed cell imaging, cells were fixed using 4% paraformaldehyde (PFA), permeabilized with 0.2% Triton-X, and stained with 1× DAPI (4′,6-diamidino-2-phenylindole) to visualize nuclear DNA. For the percent of cells infected vs uninfected, nuclei within YFP(−) and mCherry(+) (infected) DRSCs were counted and compared to the total number of nuclei counted in three images from each condition and averaged. For the Lenacapavir dose titrations, DRSCs were infected with NL4-3 HIV-1 virus at an MOI of 1 and fixed with 4%PFA 24 hpi for imaging and analysis. Lenacapavir (MedChemExpress, Cat. HY-111964) was added at the indicated doses during addition of the virus.

### Western blots

DRSCs were lysed with cracking buffer (8 M urea, 5% SDS, 40 mM Tris 7.0, 0.1 mM EDTA, 25% glycerol, bromophenol blue). The amount of lysate loaded in each well was normalized based on GAPDH levels. Lysates were run using Mini PROTEAN TGX precast gels (Bio-Rad, Cat# 4569035) and transferred to a PVDF transfer membrane (Immobilon, Millipore, Cat# 1PFL00010) using a Power Blotter XL semi-dry transfer system (Invitrogen). Membranes were incubated overnight at RT in Intercept blocking buffer (LI-COR, Cat# 927-60001), 1 h at RT for primary antibodies, and 40 min at RT for secondary antibodies. Antibodies were diluted in a blocking buffer. Antibodies used were anti-APOBEC3G (Cell Signaling, Cat# D96CZ, 1:1,500), anti-GAPDH (Santa Cruz Biotechnology, Cat# sc-32233, 1:1,000), and anti-HIV-1 p24 (made in house, 1:150). The membrane was then imaged using a LI-COR Odyssey Fc with images analyzed using Image Studio Lite software.

Cellpose (https://www.cellpose.org/) was used to segment individual cells through the Gag-mCherry signal in the cytoplasm of infected DRSCs, utilizing the Cytoplasm 2.0 pre-trained model with a cell diameter of 30 microns while simplifying contours. The resulting spots were exported as ROIs to ImageJ’s ROI manager, where fluorescence measurements were made on the YFP channel for each cell ROI. To measure cell morphology, we used the FIJI plug-in Stardist (https://imagej.net/plugins/stardist) to segment the round cells from their fluorescent signals and proceeded to use that image processing tool to create regions of interest around cells and measure their circularity. The formula for circularity is circularity = 4pi(area/perimeter^2^), so as the value reaches closer to 1, the closer the object measured is to a perfect circle. We determined that Vif-arrested cells had a circularity of 0.96 and above by confirming arrest in analyzed cells within one of our NL4-3 Vif infection movies, so we set that circularity limit along with a minimal area size requirement as our threshold for detecting if a cell is arrested.

### Flow cytometry

Infected and uninfected cells were washed with 1× PBS, trypsinized, and then fixed with 2% PFA (4% PFA for un-attenuated infection cells) in preparation for flow cytometry analysis. For co-receptor level analysis, our panel of co-receptor DRSCs was incubated with conjugated antibodies for CCR5 (PE anti-human CD195 (CCR5), cat. 313707 BioLegend) and CXCR4 (APC anti-human CD184 (CXCR4), cat. 306509 BioLegend) for 30 min at 4°C. Cells were sorted using a BD FACSAria Cell Sorter at the UW-Madison Flow Cytometry Laboratory and results were analyzed with FlowJo software.

### Statistical analyses

The quantified results represent averages from three independent fields of view for live cell imaging analyses and three independent experiments for all other tests. Statistical significance was calculated using the Student’s t test or one-way analysis of variance (ANOVA) as required based on the number of conditions compared and was defined as *P* ≤ 0.05. GraphPad Prism was used for statistical analysis and graphing of results.

## Data Availability

Any data generated from the experiments described in this article but not included in the figures are available from the corresponding author (N.M.S.) upon request.

## SUPPLEMENTAL MATERIAL

Supplementa legends jvi.01579-24-s0001.docx Legends for Movies S1 to S3.

Movie S1 jvi.01579-24-s0002.avi DRSC activation.

Movie S2 jvi.01579-24-s0003.avi DRSCs infected at high or low MOI.

Movie S3 jvi.01579-24-s0004.avi B3 and C1 Vif demonstrate defective anti-A3G activity.

ASM does not own the copyrights to Supplemental Material that may be linked to, or accessed through, an article. The authors have granted ASM a non-exclusive, world-wide license to publish the Supplemental Material files. Please contact the corresponding author directly for reuse.

## References

[1] Hu W-S, Rhodes T, Dang Q, Pathak V. 2003. Retroviral recombination review of genetic analyses. Front Biosci 8:d143–155. doi:10.2741/940 12456341

[2] Smyth RP, Davenport MP, Mak J. 2012. The origin of genetic diversity in HIV-1. Virus Res 169:415–429. doi:10.1016/j.virusres.2012.06.015 22728444

[3] Coffin J, Swanstrom R. 2013. HIV pathogenesis: dynamics and genetics of viral populations and infected cells. Cold Spring Harb Perspect Med 3:a012526. doi:10.1101/cshperspect.a012526 23284080 PMC3530041

[4] Sanjuán R, Domingo-Calap P. 2021. Genetic diversity and evolution of viral populations. Encycl Virol 53–61. doi:10.1016/B978-0-12-809633-8.20958-8

[5] Domingo E. 2016. Molecular basis of genetic variation of viruses. Virus Popul:35–71. doi:10.1016/B978-0-12-800837-9.00002-2

[6] Renjifo B, Gilbert P, Chaplin B, Msamanga G, Mwakagile D, Fawzi W, Essex M. 2004. Preferential in-utero transmission of HIV-1 subtype C as compared to HIV-1 subtype A or D. AIDS 18:1629–1636. doi:10.1097/01.aids.0000131392.68597.34 15280773

[7] John-Stewart GC, Nduati RW, Rousseau CM, Mbori-Ngacha DA, Richardson BA, Rainwater S, Panteleeff DD, Overbaugh J. 2005. Subtype C Is associated with increased vaginal shedding of HIV-1. J Infect Dis 192:492–496. doi:10.1086/431514 15995964 PMC3387274

[8] Hudgens MG, Longini IM Jr, Vanichseni S, Hu DJ, Kitayaporn D, Mock PA, Halloran ME, Satten GA, Choopanya K, Mastro TD. 2002. Subtype-specific transmission probabilities for human immunodeficiency virus type 1 among injecting drug users in Bangkok, Thailand. Am J Epidemiol 155:159–168. doi:10.1093/aje/155.2.159 11790680

[9] Taylor BS, Sobieszczyk ME, McCutchan FE, Hammer SM. 2008. The challenge of HIV-1 subtype diversity. N Engl J Med 358:1590–1602. doi:10.1056/NEJMra0706737 18403767 PMC2614444

[10] Ariën KK, Abraha A, Quiñones-Mateu ME, Kestens L, Vanham G, Arts EJ. 2005. The replicative fitness of primary human immunodeficiency virus type 1 (HIV-1) group M, HIV-1 group O, and HIV-2 isolates. J Virol 79:8979–8990. doi:10.1128/JVI.79.14.8979-8990.2005 15994792 PMC1168791

[11] Marozsan AJ, Moore DM, Lobritz MA, Fraundorf E, Abraha A, Reeves JD, Arts EJ. 2005. Differences in the fitness of two diverse wild-type human immunodeficiency virus type 1 isolates are related to the efficiency of cell binding and entry. J Virol 79:7121–7134. doi:10.1128/JVI.79.11.7121-7134.2005 15890952 PMC1112120

[12] Kanki PJ, Hamel DJ, Sankalé JL, Hsieh C c, Thior I, Barin F, Woodcock SA, Guèye-Ndiaye A, Zhang E, Montano M, Siby T, Marlink R, NDoye I, Essex ME, MBoup S. 1999. Human immunodeficiency virus type 1 subtypes differ in disease progression. J Infect Dis 179:68–73. doi:10.1086/314557 9841824

[13] Baeten JM, Chohan B, Lavreys L, Chohan V, McClelland RS, Certain L, Mandaliya K, Jaoko W, Overbaugh J. 2007. HIV-1 subtype D infection is associated with faster disease progression than subtype A in spite of similar plasma HIV-1 loads. J Infect Dis 195:1177–1180. doi:10.1086/512682 17357054

[14] Kaleebu P, Ross A, Morgan D, Yirrell D, Oram J, Rutebemberwa A, Lyagoba F, Hamilton L, Biryahwaho B, Whitworth J. 2001. Relationship between HIV-1 Env subtypes A and D and disease progression in A rural Ugandan cohort. AIDS 15:293–299. doi:10.1097/00002030-200102160-00001 11273208

[15] Kaleebu P, French N, Mahe C, Yirrell D, Watera C, Lyagoba F, Nakiyingi J, Rutebemberwa A, Morgan D, Weber J, Gilks C, Whitworth J. 2002. Effect of human immunodeficiency virus (HIV) type 1 envelope subtypes A and D on disease progression in A large cohort of HIV-1-positive persons in Uganda. J Infect Dis 185:1244–1250. doi:10.1086/340130 12001041

[16] Kiwanuka N, Laeyendecker O, Robb M, Kigozi G, Arroyo M, McCutchan F, Eller LA, Eller M, Makumbi F, Birx D, Wabwire-Mangen F, Serwadda D, Sewankambo NK, Quinn TC, Wawer M, Gray R. 2008. Effect of human immunodeficiency virus type 1 (HIV-1) subtype on disease progression in persons from Rakai, Uganda, with incident HIV-1 infection. J Infect Dis 197:707–713. doi:10.1086/527416 18266607

[17] Vasan A, Renjifo B, Hertzmark E, Chaplin B, Msamanga G, Essex M, Fawzi W, Hunter D. 2006. Different rates of disease progression of HIV type 1 infection in Tanzania based on infecting subtype. Clin Infect Dis 42:843–852. doi:10.1086/499952 16477563

[18] Sacktor N, Nakasujja N, Skolasky RL, Rezapour M, Robertson K, Musisi S, Katabira E, Ronald A, Clifford DB, Laeyendecker O, Quinn TC. 2009. HIV subtype D is associated with dementia, compared with subtype A, in immunosuppressed individuals at risk of cognitive impairment in Kampala, Uganda. Clin Infect Dis 49:780–786. doi:10.1086/605284 19622045 PMC2941149

[19] Hemelaar J, Elangovan R, Yun J, Dickson-Tetteh L, Fleminger I, Kirtley S, Williams B, Gouws-Williams E, Ghys PD, WHO–UNAIDS Network for HIV Isolation Characterisation. 2019. Global and regional molecular epidemiology of HIV-1, 1990-2015: a systematic review, global survey, and trend analysis. Lancet Infect Dis 19:143–155. doi:10.1016/S1473-3099(18)30647-9 30509777

[20] Williams A, Menon S, Crowe M, Agarwal N, Biccler J, Bbosa N, Ssemwanga D, Adungo F, Moecklinghoff C, Macartney M, Oriol-Mathieu V. 2023. Geographic and population distributions of human immunodeficiency virus (HIV)–1 and HIV-2 circulating subtypes: a systematic literature review and meta-analysis (2010–2021). J Infect Dis 228:1583–1591. doi:10.1093/infdis/jiad327 37592824 PMC10681860

[21] Berger EA, Doms RW, Fenyö EM, Korber BT, Littman DR, Moore JP, Sattentau QJ, Schuitemaker H, Sodroski J, Weiss RA. 1998. A new classification for HIV-1. Nature New Biol 391:240. doi:10.1038/34571 9440686

[22] Hemelaar J, Gouws E, Ghys PD, Osmanov S. 2011. WHO-UNAIDS network for HIV isolation and characterisation. global trends in molecular epidemiology of HIV-1 during 2000-2007. AIDS Lond Engl 25:679–689. doi:10.1097/QAD.0b013e328342ff93 PMC3755761 21297424

[23] Li G, Piampongsant S, Faria NR, Voet A, Pineda-Peña A-C, Khouri R, Lemey P, Vandamme A-M, Theys K. 2015. An integrated map of HIV genome-wide variation from a population perspective. Retrovirology (Auckl) 12:18. doi:10.1186/s12977-015-0148-6 PMC4358901 25808207

[24] Wei X, Decker JM, Wang S, Hui H, Kappes JC, Wu X, Salazar-Gonzalez JF, Salazar MG, Kilby JM, Saag MS, Komarova NL, Nowak MA, Hahn BH, Kwong PD, Shaw GM. 2003. Antibody neutralization and escape by HIV-1. Nature New Biol 422:307–312. doi:10.1038/nature01470 12646921

[25] Crispin M, Ward AB, Wilson IA. 2018. Structure and immune recognition of the HIV glycan shield. Annu Rev Biophys 47:499–523. doi:10.1146/annurev-biophys-060414-034156 29595997 PMC6163090

[26] Seabright GE, Doores KJ, Burton DR, Crispin M. 2019. Protein and glycan mimicry in HIV vaccine design. J Mol Biol 431:2223–2247. doi:10.1016/j.jmb.2019.04.016 31028779 PMC6556556

[27] Hunter JR, dos Santos DEM, Munerato P, Janini LM, Castelo A, Sucupira MC, Truong H-HM, Diaz RS. 2022. Antiretroviral drug-resistance mutations on the gag gene: mutation dynamics during analytic treatment interruption among individuals experiencing virologic failure. Pathogens 11:534. doi:10.3390/pathogens11050534 35631055 PMC9145614

[28] Cilento ME, Kirby KA, Sarafianos SG. 2021. Avoiding drug resistance in HIV reverse transcriptase. Chem Rev 121:3271–3296. doi:10.1021/acs.chemrev.0c00967 33507067 PMC8149104

[29] Xiao MA, Cleyle J, Yoo S, Forrest M, Krullaars Z, Pham HT, Mesplède T. 2023. The G118R plus R263K combination of integrase mutations associated with dolutegravir-based treatment failure reduces HIV-1 replicative capacity and integration. Antimicrob Agents Chemother 67:e0138622. doi:10.1128/aac.01386-22 37071019 PMC10190594

[30] Jayaraman B, Fernandes JD, Yang S, Smith C, Frankel AD. 2019. Highly mutable linker regions regulate HIV-1 rev function and stability. Sci Rep 9:5139. doi:10.1038/s41598-019-41582-7 30914719 PMC6435700

[31] Dzhivhuho G, Holsey J, Honeycutt E, O’Farrell H, Rekosh D, Hammarskjold M-L, Jackson PEH. 2022. HIV-1 Rev-RRE functional activity in primary isolates is highly dependent on minimal context-dependent changes in Rev. Sci Rep 12:18416. doi:10.1038/s41598-022-21714-2 36319640 PMC9626594

[32] Ranga U, Shankarappa R, Siddappa NB, Ramakrishna L, Nagendran R, Mahalingam M, Mahadevan A, Jayasuryan N, Satishchandra P, Shankar SK, Prasad VR. 2004. Tat protein of human immunodeficiency virus type 1 subtype C strains is a defective chemokine. J Virol 78:2586–2590. doi:10.1128/jvi.78.5.2586-2590.2004 14963162 PMC369202

[33] Rossenkhan R, MacLeod IJ, Sebunya TK, Castro-Nallar E, McLane MF, Musonda R, Gashe BA, Novitsky V, Essex M. 2013. Tat Exon 1 exhibits functional diversity during HIV-1 subtype C primary infection. J Virol 87:5732–5745. doi:10.1128/JVI.03297-12 23487450 PMC3648179

[34] Roy CN, Khandaker I, Oshitani H. 2015. intersubtype genetic variation of HIV-1 tat exon 1. AIDS Res Hum Retroviruses 31:641–648. doi:10.1089/aid.2014.0346 25748226

[35] Roy CN, Khandaker I, Oshitani H. 2015. Evolutionary dynamics of Tat in HIV-1 subtypes B and C. PLoS One 10:e0129896. doi:10.1371/journal.pone.0129896 26087118 PMC4472691

[36] Spector C, Mele AR, Wigdahl B, Nonnemacher MR. 2019. Genetic variation and function of the HIV-1 Tat protein. Med Microbiol Immunol 208:131–169. doi:10.1007/s00430-019-00583-z 30834965 PMC6476422

[37] Simon V, Zennou V, Murray D, Huang Y, Ho DD, Bieniasz PD. 2005. Natural variation in Vif: differential impact on APOBEC3G/3F and a potential role in HIV-1 diversification. PLoS Pathog 1:e6. doi:10.1371/journal.ppat.0010006 16201018 PMC1238741

[38] Iwabu Y, Kinomoto M, Tatsumi M, Fujita H, Shimura M, Tanaka Y, Ishizaka Y, Nolan D, Mallal S, Sata T, Tokunaga K. 2010. Differential anti-APOBEC3G activity of HIV-1 Vif proteins derived from different subtypes. J Biol Chem 285:35350–35358. doi:10.1074/jbc.M110.173286 20833716 PMC2975159

[39] Binka M, Ooms M, Steward M, Simon V. 2012. The activity spectrum of Vif from multiple HIV-1 subtypes against APOBEC3G, APOBEC3F, and APOBEC3H. J Virol 86:49–59. doi:10.1128/JVI.06082-11 22013041 PMC3255910

[40] Altamirano-Flores JS, Alvarado-Hernández LÁ, Cuevas-Tello JC, Tino P, Guerra-Palomares SE, Garcia-Sepulveda CA. 2023. Identification of clinically relevant HIV Vif protein motif mutations through machine learning and undersampling. Cells 12:772. doi:10.3390/cells12050772 36899908 PMC10001277

[41] Lum JJ, Cohen OJ, Nie Z, Weaver JG, Gomez TS, Yao X-J, Lynch D, Pilon AA, Hawley N, Kim JE, Chen Z, Montpetit M, Sanchez-Dardon J, Cohen EA, Badley AD. 2003. Vpr R77Q is associated with long-term nonprogressive HIV infection and impaired induction of apoptosis. J Clin Invest 111:1547–1554. doi:10.1172/JCI16233 12750404 PMC155040

[42] Dampier W, Antell GC, Aiamkitsumrit B, Nonnemacher MR, Jacobson JM, Pirrone V, Zhong W, Kercher K, Passic S, Williams JW, James T, Devlin KN, Giovannetti T, Libon DJ, Szep Z, Ehrlich GD, Wigdahl B, Krebs FC. 2017. Specific amino acids in HIV-1 Vpr are significantly associated with differences in patient neurocognitive status. J Neurovirol 23:113–124. doi:10.1007/s13365-016-0462-3 27400931 PMC5226925

[43] Pickering S, Hué S, Kim E-Y, Reddy S, Wolinsky SM, Neil SJD. 2014. Preservation of tetherin and CD4 counter-activities in circulating Vpu alleles despite extensive sequence variation within HIV-1 infected individuals. PLoS Pathog 10:e1003895. doi:10.1371/journal.ppat.1003895 24465210 PMC3900648

[44] Soares R, Rocha G, Nogueira C, Meliço-Silvestre A, Gonçalves T. 2014. R77Q and Q3R HIV1-VPR mutations in an otherwise asymptomatic 5-year-old child with repeated ear infections. JMM Case Rep 1:e002709. doi:10.1099/jmmcr.0.002709 28663807 PMC5415930

[45] Kruize Z, van Nuenen AC, van Wijk SW, Girigorie AF, van Dort KA, Booiman T, Kootstra NA. 2021. Nef obtained from individuals with HIV-1 vary in their ability to antagonize SERINC3- and SERINC5-mediated HIV-1 restriction. Viruses 13:423. doi:10.3390/v13030423 33800773 PMC8000780

[46] Kalu AW, Telele NF, Gebreselasie S, Fekade D, Abdurahman S, Marrone G, Sönnerborg A. 2017. Prediction of coreceptor usage by five bioinformatics tools in a large Ethiopian HIV-1 subtype C cohort. PLoS One 12:e0182384. doi:10.1371/journal.pone.0182384 28841646 PMC5571954

[47] Pai NP, Karellis A, Kim J, Peter T. 2020. Modern diagnostic technologies for HIV. Lancet HIV 7:e574–e581. doi:10.1016/S2352-3018(20)30190-9 32763220

[48] Henningsson R, Moratorio G, Bordería AV, Vignuzzi M, Fontes M. 2019. DISSEQT-DIStribution-based modeling of SEQuence space time dynamics. Virus Evol 5:vez028. doi:10.1093/ve/vez028 31392032 PMC6680062

[49] Jiang C, Lian X, Gao C, Sun X, Einkauf KB, Chevalier JM, Chen SMY, Hua S, Rhee B, Chang K, et al.. 2020. Distinct viral reservoirs in individuals with spontaneous control of HIV-1. Nature New Biol 585:261–267. doi:10.1038/s41586-020-2651-8 PMC7837306 32848246

[50] Einkauf KB, Lee GQ, Gao C, Sharaf R, Sun X, Hua S, Chen SM, Jiang C, Lian X, Chowdhury FZ, Rosenberg ES, Chun T-W, Li JZ, Yu XG, Lichterfeld M. 2019. Intact HIV-1 proviruses accumulate at distinct chromosomal positions during prolonged antiretroviral therapy. J Clin Invest 129:988–998. doi:10.1172/JCI124291 30688658 PMC6391088

[51] Kimpton J, Emerman M. 1992. Detection of replication-competent and pseudotyped human immunodeficiency virus with a sensitive cell line on the basis of activation of an integrated beta-galactosidase gene. J Virol 66:2232–2239. doi:10.1128/JVI.66.4.2232-2239.1992 1548759 PMC289016

[52] Schwartz O, Virelizier JL, Montagnier L, Hazan U. 1990. A microtransfection method using the luciferase-encoding reporter gene for the assay of human immunodeficiency virus LTR promoter activity. Gene 88:197–205. doi:10.1016/0378-1119(90)90032-m 2189784

[53] Gervaix A, West D, Leoni LM, Richman DD, Wong-Staal F, Corbeil J. 1997. A new reporter cell line to monitor HIV infection and drug susceptibility in vitro. Proc Natl Acad Sci U S A 94:4653–4658. doi:10.1073/pnas.94.9.4653 9114046 PMC20779

[54] Mörner A, Björndal A, Albert J, Kewalramani VN, Littman DR, Inoue R, Thorstensson R, Fenyö EM, Björling E. 1999. Primary human immunodeficiency virus type 2 (HIV-2) isolates, like HIV-1 isolates, frequently use CCR5 but show promiscuity in coreceptor usage. J Virol 73:2343–2349. doi:10.1128/JVI.73.3.2343-2349.1999 9971817 PMC104479

[55] Wei X, Decker JM, Liu H, Zhang Z, Arani RB, Kilby JM, Saag MS, Wu X, Shaw GM, Kappes JC. 2002. Emergence of resistant human immunodeficiency virus type 1 in patients receiving fusion inhibitor (T-20) monotherapy. Antimicrob Agents Chemother 46:1896–1905. doi:10.1128/AAC.46.6.1896-1905.2002 12019106 PMC127242

[56] Sheehy AM, Gaddis NC, Choi JD, Malim MH. 2002. Isolation of a human gene that inhibits HIV-1 infection and is suppressed by the viral Vif protein. Nature New Biol 418:646–650. doi:10.1038/nature00939 12167863

[57] Sheehy AM, Gaddis NC, Malim MH. 2003. The antiretroviral enzyme APOBEC3G is degraded by the proteasome in response to HIV-1 Vif. Nat Med 9:1404–1407. doi:10.1038/nm945 14528300

[58] Zhang H, Yang B, Pomerantz RJ, Zhang C, Arunachalam SC, Gao L. 2003. The cytidine deaminase CEM15 induces hypermutation in newly synthesized HIV-1 DNA. Nature New Biol 424:94–98. doi:10.1038/nature01707 PMC1350966 12808465

[59] Stopak K, de Noronha C, Yonemoto W, Greene WC. 2003. HIV-1 Vif blocks the antiviral activity of APOBEC3G by impairing both its translation and intracellular stability. Mol Cell 12:591–601. doi:10.1016/S1097-2765(03)00353-8 14527406

[60] Marin M, Rose KM, Kozak SL, Kabat D. 2003. HIV-1 Vif protein binds the editing enzyme APOBEC3G and induces its degradation. Nat Med 9:1398–1403. doi:10.1038/nm946 14528301

[61] Vartanian JP, Meyerhans A, Asjö B, Wain-Hobson S. 1991. Selection, recombination, and G----A hypermutation of human immunodeficiency virus type 1 genomes. J Virol 65:1779–1788. doi:10.1128/JVI.65.4.1779-1788.1991 2002543 PMC239985

[62] Yu X, Yu Y, Liu B, Luo K, Kong W, Mao P, Yu X-F. 2003. Induction of APOBEC3G ubiquitination and degradation by an HIV-1 Vif-Cul5-SCF complex. Science 302:1056–1060. doi:10.1126/science.1089591 14564014

[63] Mehle A, Strack B, Ancuta P, Zhang C, McPike M, Gabuzda D. 2004. Vif overcomes the innate antiviral activity of APOBEC3G by promoting its degradation in the ubiquitin-proteasome pathway. J Biol Chem 279:7792–7798. doi:10.1074/jbc.M313093200 14672928

[64] Holmes M, Zhang F, Bieniasz PD. 2015. Single-cell and single-cycle analysis of HIV-1 replication. PLoS Pathog 11:e1004961. doi:10.1371/journal.ppat.1004961 26086614 PMC4472667

[65] Karn J, Stoltzfus CM. 2012. Transcriptional and posttranscriptional regulation of HIV-1 gene expression. Cold Spring Harb Perspect Med 2:a006916. doi:10.1101/cshperspect.a006916 22355797 PMC3281586

[66] Pollard VW, Malim MH. 1998. The HIV-1 rev protein. Annu Rev Microbiol 52:491–532. doi:10.1146/annurev.micro.52.1.491 9891806

[67] Cecilia D, KewalRamani VN, O’Leary J, Volsky B, Nyambi P, Burda S, Xu S, Littman DR, Zolla-Pazner S. 1998. Neutralization profiles of primary human immunodeficiency virus type 1 isolates in the context of coreceptor usage. J Virol 72:6988–6996. doi:10.1128/JVI.72.9.6988-6996.1998 9696790 PMC109918

[68] Hosseini I, Mac Gabhann F. 2012. Multi-scale modeling of HIV infection in vitro and APOBEC3G-based anti-retroviral therapy. PLoS Comput Biol 8:e1002371. doi:10.1371/journal.pcbi.1002371 22346743 PMC3276540

[69] Salamango DJ, McCann JL, Demir Ö, Becker JT, Wang J, Lingappa JR, Temiz NA, Brown WL, Amaro RE, Harris RS. 2020. Functional and structural insights into a Vif/PPP2R5 complex elucidated using patient HIV-1 isolates and computational modeling. J Virol 94:e00631-20. doi:10.1128/JVI.00631-20 32847850 PMC7565612

[70] Becker JT, Edward L, Evans III, Bayleigh E. 2019. HIV-1 genome trafficking initiates APOBEC3G packaging in the cytosol. bioRxiv. doi:10.1101/846105

[71] Evans EL, Becker JT, Fricke SL, Patel K, Sherer NM. 2018. HIV-1 vif’s capacity to manipulate the cell cycle is species specific. J Virol 92:e02102-17. doi:10.1128/JVI.02102-17 29321323 PMC5972884

[72] Gallois-Montbrun S, Kramer B, Swanson CM, Byers H, Lynham S, Ward M, Malim MH. 2007. Antiviral protein APOBEC3G localizes to ribonucleoprotein complexes found in P bodies and stress granules. J Virol 81:2165–2178. doi:10.1128/JVI.02287-06 17166910 PMC1865933

[73] Sundquist WI, Krausslich H-G. 2012. HIV-1 assembly, budding, and maturation. Cold Spring Harb Perspect Med 2:a006924–a006924. doi:10.1101/cshperspect.a006924 22762019 PMC3385941

[74] Freed EO. 2015. HIV-1 assembly, release and maturation. Nat Rev Microbiol 13:484–496. doi:10.1038/nrmicro3490 26119571 PMC6936268

[75] Tang C, Loeliger E, Luncsford P, Kinde I, Beckett D, Summers MF. 2004. Entropic switch regulates myristate exposure in the HIV-1 matrix protein. Proc Natl Acad Sci U S A 101:517–522. doi:10.1073/pnas.0305665101 14699046 PMC327179

[76] Ono A, Ablan SD, Lockett SJ, Nagashima K, Freed EO. 2004. Phosphatidylinositol (4,5) bisphosphate regulates HIV-1 Gag targeting to the plasma membrane. Proc Natl Acad Sci U S A 101:14889–14894. doi:10.1073/pnas.0405596101 15465916 PMC522033

[77] Kutluay SB, Bieniasz PD. 2010. Analysis of the initiating events in HIV-1 particle assembly and genome packaging. PLoS Pathog 6:e1001200. doi:10.1371/journal.ppat.1001200 21124996 PMC2987827

[78] Link JO, Rhee MS, Tse WC, Zheng J, Somoza JR, Rowe W, Begley R, Chiu A, Mulato A, Hansen D, et al.. 2020. Clinical targeting of HIV capsid protein with a long-acting small molecule. Nature New Biol 584:614–618. doi:10.1038/s41586-020-2443-1 PMC8188729 32612233

[79] Sanjuán R, Domingo-Calap P. 2016. Mechanisms of viral mutation. Cell Mol Life Sci 73:4433–4448. doi:10.1007/s00018-016-2299-6 27392606 PMC5075021

[80] Peck KM, Lauring AS. 2018. Complexities of viral mutation rates. J Virol 92:e01031-17. doi:10.1128/JVI.01031-17 29720522 PMC6026756

[81] Schröfelbauer B, Senger T, Manning G, Landau NR. 2006. Mutational alteration of human immunodeficiency virus type 1 Vif allows for functional interaction with nonhuman primate APOBEC3G. J Virol 80:5984–5991. doi:10.1128/JVI.00388-06 16731937 PMC1472613

[82] Mehle A, Wilson H, Zhang C, Brazier AJ, McPike M, Pery E, Gabuzda D. 2007. Identification of an APOBEC3G binding site in human immunodeficiency virus type 1 Vif and inhibitors of Vif-APOBEC3G binding. J Virol 81:13235–13241. doi:10.1128/JVI.00204-07 17898068 PMC2169136

[83] Mehle A., Thomas ER, Rajendran KS, Gabuzda D. 2006. A zinc-binding region in Vif binds Cul5 and determines cullin selection. J Biol Chem 281:17259–17265. doi:10.1074/jbc.M602413200 16636053

[84] Marin M, Golem S, Rose KM, Kozak SL, Kabat D. 2008. Human immunodeficiency virus type 1 Vif functionally interacts with diverse APOBEC3 cytidine deaminases and moves with them between cytoplasmic sites of mRNA metabolism. J Virol 82:987–998. doi:10.1128/JVI.01078-07 17977970 PMC2224600

[85] Baig TT, Feng Y, Chelico L. 2014. Determinants of efficient degradation of APOBEC3 restriction factors by HIV-1 Vif. J Virol 88:14380–14395. doi:10.1128/JVI.02484-14 25275135 PMC4249154

[86] Zhao K, Du J, Rui Y, Zheng W, Kang J, Hou J, Wang K, Zhang W, Simon VA, Yu X-F. 2015. Evolutionarily conserved pressure for the existence of distinct G2/M cell cycle arrest and A3H inactivation functions in HIV-1 Vif. Cell Cycle 14:838–847. doi:10.1080/15384101.2014.1000212 25590520 PMC4612454

[87] Stringer C, Wang T, Michaelos M, Pachitariu M. 2021. Cellpose: a generalist algorithm for cellular segmentation. Nat Methods 18:100–106. doi:10.1038/s41592-020-01018-x 33318659

[88] Greenwood EJ, Matheson NJ, Wals K, van den Boomen DJ, Antrobus R, Williamson JC, Lehner PJ. 2016. Temporal proteomic analysis of HIV infection reveals remodelling of the host phosphoproteome by lentiviral Vif variants. Elife 5:e18296. doi:10.7554/eLife.18296 27690223 PMC5085607

[89] Nilsson J. 2019. Protein phosphatases in the regulation of mitosis. J Cell Biol 218:395–409. doi:10.1083/jcb.201809138 30446607 PMC6363451

[90] Salamango DJ, Ikeda T, Moghadasi SA, Wang J, McCann JL, Serebrenik AA, Ebrahimi D, Jarvis MC, Brown WL, Harris RS. 2019. HIV-1 Vif triggers cell cycle arrest by degrading cellular PPP2R5 phospho-regulators. Cell Rep 29:1057–1065. doi:10.1016/j.celrep.2019.09.057 31665623 PMC6903395

[91] Izumi T, Io K, Matsui M, Shirakawa K, Shinohara M, Nagai Y, Kawahara M, Kobayashi M, Kondoh H, Misawa N, Koyanagi Y, Uchiyama T, Takaori-Kondo A. 2010. HIV-1 viral infectivity factor interacts with TP53 to induce G2 cell cycle arrest and positively regulate viral replication. Proc Natl Acad Sci U S A 107:20798–20803. doi:10.1073/pnas.1008076107 21071676 PMC2996458

[92] Zhao Y, Fu C, Zhang W, Ye C, Wang Z, Ma H. 2022. Automatic segmentation of cervical cells based on star-convex polygons in pap smear images. Bioengineering (Basel) 10:47. doi:10.3390/bioengineering10010047 36671619 PMC9854569

[93] Ghone D, Evans EL III, Bandini M, Stephenson KG, Sherer NM, Suzuki A. 2024. HIV-1 Vif disrupts phosphatase feedback regulation at the kinetochore, leading to a pronounced pseudo-metaphase arrest. Microbiology. doi:10.1101/2024.07.30.605839 PMC11906157 40080415

[94] Wong HT, Luperchio AM, Riley S, Salamango DJ. 2023. Inhibition of ATM-directed antiviral responses by HIV-1 Vif. PLoS Pathog 19:e1011634. doi:10.1371/journal.ppat.1011634 37669285 PMC10503699

[95] Klatzmann D, Champagne E, Chamaret S, Gruest J, Guetard D, Hercend T, Gluckman J-C, Montagnier L. 1984. T-lymphocyte T4 molecule behaves as the receptor for human retrovirus LAV. Nature New Biol 312:767–768. doi:10.1038/312767a0 6083454

[96] Dalgleish AG, Beverley PC, Clapham PR, Crawford DH, Greaves MF, Weiss RA. 1984. The CD4 (T4) antigen is an essential component of the receptor for the AIDS retrovirus. Nature New Biol 312:763–767. doi:10.1038/312763a0 6096719

[97] Alkhatib G, Combadiere C, Broder CC, Feng Y, Kennedy PE, Murphy PM, Berger EA. 1996. CC CKR5: a RANTES, MIP-1alpha, MIP-1beta receptor as a fusion cofactor for macrophage-tropic HIV-1. Science 272:1955–1958. doi:10.1126/science.272.5270.1955 8658171

[98] Choe H, Farzan M, Sun Y, Sullivan N, Rollins B, Ponath PD, Wu L, Mackay CR, LaRosa G, Newman W, Gerard N, Gerard C, Sodroski J. 1996. The beta-chemokine receptors CCR3 and CCR5 facilitate infection by primary HIV-1 isolates. Cell 85:1135–1148. doi:10.1016/s0092-8674(00)81313-6 8674119

[99] Deng H, Liu R, Ellmeier W, Choe S, Unutmaz D, Burkhart M, Di Marzio P, Marmon S, Sutton RE, Hill CM, Davis CB, Peiper SC, Schall TJ, Littman DR, Landau NR. 1996. Identification of a major co-receptor for primary isolates of HIV-1. Nature New Biol 381:661–666. doi:10.1038/381661a0 8649511

[100] Feng Y, Broder CC, Kennedy PE, Berger EA. 1996. HIV-1 entry cofactor: functional cDNA cloning of a seven-transmembrane, G protein-coupled receptor. Science 272:872–877. doi:10.1126/science.272.5263.872 8629022

[101] Oberlin E, Amara A, Bachelerie F, Bessia C, Virelizier JL, Arenzana-Seisdedos F, Schwartz O, Heard JM, Clark-Lewis I, Legler DF, Loetscher M, Baggiolini M, Moser B. 1996. The CXC chemokine SDF-1 is the ligand for LESTR/fusin and prevents infection by T-cell-line-adapted HIV-1. Nature New Biol 382:833–835. doi:10.1038/382833a0 8752281

[102] Connor RI, Sheridan KE, Ceradini D, Choe S, Landau NR. 1997. Change in coreceptor use correlates with disease progression in HIV-1--infected individuals. J Exp Med 185:621–628. doi:10.1084/jem.185.4.621 9034141 PMC2196142

[103] Doranz BJ, Rucker J, Yi Y, Smyth RJ, Samson M, Peiper SC, Parmentier M, Collman RG, Doms RW. 1996. A dual-tropic primary HIV-1 isolate that uses fusin and the beta-chemokine receptors CKR-5, CKR-3, and CKR-2b as fusion cofactors. Cell 85:1149–1158. doi:10.1016/s0092-8674(00)81314-8 8674120

[104] Schuitemaker H, Koot M, Kootstra NA, Dercksen MW, de Goede RE, van Steenwijk RP, Lange JM, Schattenkerk JK, Miedema F, Tersmette M. 1992. Biological phenotype of human immunodeficiency virus type 1 clones at different stages of infection: progression of disease is associated with a shift from monocytotropic to T-cell-tropic virus population. J Virol 66:1354–1360. doi:10.1128/JVI.66.3.1354-1360.1992 1738194 PMC240857

[105] Lin NH, Kuritzkes DR. 2009. Tropism testing in the clinical management of HIV-1 infection. Curr Opin HIV AIDS 4:481–487. doi:10.1097/COH.0b013e328331b929 20048714 PMC2874683

[106] Huang W, Eshleman SH, Toma J, Fransen S, Stawiski E, Paxinos EE, Whitcomb JM, Young AM, Donnell D, Mmiro F, Musoke P, Guay LA, Jackson JB, Parkin NT, Petropoulos CJ. 2007. Coreceptor tropism in human immunodeficiency virus type 1 subtype D: high prevalence of CXCR4 tropism and heterogeneous composition of viral populations. J Virol 81:7885–7893. doi:10.1128/JVI.00218-07 17507467 PMC1951291

[107] Riemenschneider M, Cashin KY, Budeus B, Sierra S, Shirvani-Dastgerdi E, Bayanolhagh S, Kaiser R, Gorry PR, Heider D. 2016. Genotypic prediction of co-receptor tropism of HIV-1 subtypes A and C. Sci Rep 6:24883. doi:10.1038/srep24883 27126912 PMC4850382

[108] Löchel HF, Riemenschneider M, Frishman D, Heider D. 2018. SCOTCH: subtype A coreceptor tropism classification in HIV-1. Bioinformatics 34:2575–2580. doi:10.1093/bioinformatics/bty170 29554213

[109] Montefiori DC. 2009. Measuring HIV neutralization in a luciferase reporter gene assay. Methods Mol. Biol. Clifton NJ 485:395–405. doi:10.1007/978-1-59745-170-3_26 19020839

[110] Ganor Y, Real F, Sennepin A, Dutertre C-A, Prevedel L, Xu L, Tudor D, Charmeteau B, Couedel-Courteille A, Marion S, Zenak A-R, Jourdain J-P, Zhou Z, Schmitt A, Capron C, Eugenin EA, Cheynier R, Revol M, Cristofari S, Hosmalin A, Bomsel M. 2019. HIV-1 reservoirs in urethral macrophages of patients under suppressive antiretroviral therapy. Nat Microbiol 4:633–644. doi:10.1038/s41564-018-0335-z 30718846

[111] Sarzotti-Kelsoe M, Bailer RT, Turk E, Lin C, Bilska M, Greene KM, Gao H, Todd CA, Ozaki DA, Seaman MS, Mascola JR, Montefiori DC. 2014. Optimization and validation of the TZM-bl assay for standardized assessments of neutralizing antibodies against HIV-1. J Immunol Methods 409:131–146. doi:10.1016/j.jim.2013.11.022 24291345 PMC4040342

[112] Sanyal A, Mailliard RB, Rinaldo CR, Ratner D, Ding M, Chen Y, Zerbato JM, Giacobbi NS, Venkatachari NJ, Patterson BK, Chargin A, Sluis-Cremer N, Gupta P. 2017. Novel assay reveals a large, inducible, replication-competent HIV-1 reservoir in resting CD4^+^ T cells. Nat Med 23:885–889. doi:10.1038/nm.4347 28553933 PMC5505781

[113] Salasc F, Gludish DW, Jarvis I, Boliar S, Wills MR, Russell DG, Lever AML, Mok H-P. 2019. A novel, sensitive dual-indicator cell line for detection and quantification of inducible, replication-competent latent HIV-1 from reservoir cells. Sci Rep 9:19325. doi:10.1038/s41598-019-55596-8 31852924 PMC6920355

[114] Saito A, Yamashita M. 2021. HIV-1 capsid variability: viral exploitation and evasion of capsid-binding molecules. Retrovirology (Auckl) 18:32. doi:10.1186/s12977-021-00577-x PMC8549334 34702294

[115] Guy B, Kieny MP, Riviere Y, Le Peuch C, Dott K, Girard M, Montagnier L, Lecocq JP. 1987. HIV F/3’ orf encodes a phosphorylated GTP-binding protein resembling an oncogene product. Nature New Biol 330:266–269. doi:10.1038/330266a0 3118220

[116] Stevenson M, Meier C, Mann AM, Chapman N, Wasiak A. 1988. Envelope glycoprotein of HIV induces interference and cytolysis resistance in CD4+ cells: mechanism for persistence in AIDS. Cell 53:483–496. doi:10.1016/0092-8674(88)90168-7 2966682 PMC9513714

[117] Van Damme N, Goff D, Katsura C, Jorgenson RL, Mitchell R, Johnson MC, Stephens EB, Guatelli J. 2008. The interferon-induced protein BST-2 restricts HIV-1 release and is downregulated from the cell surface by the viral Vpu protein. Cell Host Microbe 3:245–252. doi:10.1016/j.chom.2008.03.001 18342597 PMC2474773

[118] Willey RL, Maldarelli F, Martin MA, Strebel K. 1992. Human immunodeficiency virus type 1 Vpu protein induces rapid degradation of CD4. J Virol 66:7193–7200. doi:10.1128/JVI.66.12.7193-7200.1992 1433512 PMC240416

[119] Neil SJD, Zang T, Bieniasz PD. 2008. Tetherin inhibits retrovirus release and is antagonized by HIV-1 Vpu. Nature New Biol 451:425–430. doi:10.1038/nature06553 18200009

[120] Rosa A, Chande A, Ziglio S, De Sanctis V, Bertorelli R, Goh SL, McCauley SM, Nowosielska A, Antonarakis SE, Luban J, Santoni FA, Pizzato M. 2015. HIV-1 Nef promotes infection by excluding SERINC5 from virion incorporation. Nature New Biol 526:212–217. doi:10.1038/nature15399 PMC4861059 26416734

[121] Usami Y, Wu Y, Göttlinger HG. 2015. SERINC3 and SERINC5 restrict HIV-1 infectivity and are counteracted by Nef. Nature New Biol 526:218–223. doi:10.1038/nature15400 PMC4600458 26416733

[122] Sadler HA, Stenglein MD, Harris RS, Mansky LM. 2010. APOBEC3G contributes to HIV-1 variation through sublethal mutagenesis. J Virol 84:7396–7404. doi:10.1128/JVI.00056-10 20463080 PMC2898230

[123] Kim E-Y, Bhattacharya T, Kunstman K, Swantek P, Koning FA, Malim MH, Wolinsky SM. 2010. Human APOBEC3G-mediated editing can promote HIV-1 sequence diversification and accelerate adaptation to selective pressure. J Virol 84:10402–10405. doi:10.1128/JVI.01223-10 20660203 PMC2937764

[124] Mulder LCF, Harari A, Simon V. 2008. Cytidine deamination induced HIV-1 drug resistance. Proc Natl Acad Sci U S A 105:5501–5506. doi:10.1073/pnas.0710190105 18391217 PMC2291111

[125] Cuevas JM, Geller R, Garijo R, López-Aldeguer J, Sanjuán R. 2015. Extremely high mutation rate of HIV-1 in vivo. PLoS Biol 13:e1002251. doi:10.1371/journal.pbio.1002251 26375597 PMC4574155

[126] Dang Y, Davis RW, York IA, Zheng Y-H. 2010. Identification of 81LGxGxxIxW89 and 171EDRW174 domains from human immunodeficiency virus type 1 Vif that regulate APOBEC3G and APOBEC3F neutralizing activity. J Virol 84:5741–5750. doi:10.1128/JVI.00079-10 20335268 PMC2876610

[127] McCright B, Rivers AM, Audlin S, Virshup DM. 1996. The B56 family of protein phosphatase 2A (PP2A) regulatory subunits encodes differentiation-induced phosphoproteins that target PP2A to both nucleus and cytoplasm. J Biol Chem 271:22081–22089. doi:10.1074/jbc.271.36.22081 8703017

[128] Wang X, Bajaj R, Bollen M, Peti W, Page R. 2016. Expanding the PP2A interactome by defining a B56-specific SLiM. Structure 24:2174–2181. doi:10.1016/j.str.2016.09.010 27998540 PMC5180209

[129] Wang J, Wang Z, Yu T, Yang H, Virshup DM, Kops GJPL, Lee SH, Zhou W, Li X, Xu W, Rao Z. 2016. Crystal structure of a PP2A B56-BubR1 complex and its implications for PP2A substrate recruitment and localization. Protein Cell 7:516–526. doi:10.1007/s13238-016-0283-4 27350047 PMC4930772

[130] Maertens GN. 2016. B’-protein phosphatase 2A is a functional binding partner of delta-retroviral integrase. Nucleic Acids Res 44:364–376. doi:10.1093/nar/gkv1347 26657642 PMC4705670

[131] Kruse T, Biedenkopf N, Hertz EPT, Dietzel E, Stalmann G, López-Méndez B, Davey NE, Nilsson J, Becker S. 2018. The ebola virus nucleoprotein recruits the host PP2A-B56 phosphatase to activate transcriptional support activity of VP30. Mol Cell 69:136–145. doi:10.1016/j.molcel.2017.11.034 29290611

[132] Luperchio AM, Jónsson SR, Salamango DJ. 2022. Evolutionary conservation of PP2A antagonism and G2/M cell cycle arrest in maedi-visna virus vif. Viruses 14:1701. doi:10.3390/v14081701 36016323 PMC9413702

[133] Breuer R, Becker MS, Brechmann M, Mock T, Arnold R, Krammer PH. 2014. The protein phosphatase 2A regulatory subunit B56γ mediates suppression of T cell receptor (TCR)-induced nuclear factor-κB (NF-κB) activity. J Biol Chem 289:14996–15004. doi:10.1074/jbc.M113.533547 24719332 PMC4031550

[134] He J, Choe S, Walker R, Di Marzio P, Morgan DO, Landau NR. 1995. Human immunodeficiency virus type 1 viral protein R (Vpr) arrests cells in the G2 phase of the cell cycle by inhibiting p34cdc2 activity. J Virol 69:6705–6711. doi:10.1128/JVI.69.11.6705-6711.1995 7474080 PMC189580

[135] Garcia-Miranda P, Becker JT, Benner BE, Blume A, Sherer NM, Butcher SE. 2016. Stability of HIV frameshift site RNA correlates with frameshift efficiency and decreased virus infectivity. J Virol 90:6906–6917. doi:10.1128/JVI.00149-16 27194769 PMC4944283

[136] Adachi A, Gendelman HE, Koenig S, Folks T, Willey R, Rabson A, Martin MA. 1986. Production of acquired immunodeficiency syndrome-associated retrovirus in human and nonhuman cells transfected with an infectious molecular clone. J Virol 59:284–291. doi:10.1128/JVI.59.2.284-291.1986 3016298 PMC253077

[137] Collman R, Balliet JW, Gregory SA, Friedman H, Kolson DL, Nathanson N, Srinivasan A. 1992. An infectious molecular clone of an unusual macrophage-tropic and highly cytopathic strain of human immunodeficiency virus type 1. J Virol 66:7517–7521. doi:10.1128/JVI.66.12.7517-7521.1992 1433527 PMC240461

[138] Swanson CM, Puffer BA, Ahmad KM, Doms RW, Malim MH. 2004. Retroviral mRNA nuclear export elements regulate protein function and virion assembly. EMBO J 23:2632–2640. doi:10.1038/sj.emboj.7600270 15201866 PMC449780

[139] Pocock GM, Becker JT, Swanson CM, Ahlquist P, Sherer NM. 2016. HIV-1 and M-PMV RNA nuclear export elements program viral genomes for distinct cytoplasmic trafficking behaviors. PLoS Pathog 12:e1005565. doi:10.1371/journal.ppat.1005565 27070420 PMC4829213

[140] Gardiner JC, Mauer EJ, Sherer NM. 2016. HIV-1 gag, envelope, and extracellular determinants cooperate to regulate the stability and turnover of virological synapses. J Virol 90:6583–6597. doi:10.1128/JVI.00600-16 27170746 PMC4936141

[141] Naldini L, Blömer U, Gallay P, Ory D, Mulligan R, Gage FH, Verma IM, Trono D. 1996. In vivo gene delivery and stable transduction of nondividing cells by a lentiviral vector. Science 272:263–267. doi:10.1126/science.272.5259.263 8602510

[142] Bruce JW, Park E, Magnano C, Horswill M, Richards A, Potts G, Hebert A, Islam N, Coon JJ, Gitter A, Sherer N, Ahlquist P. 2023. HIV-1 virological synapse formation enhances infection spread by dysregulating Aurora Kinase B. PLoS Pathog 19:e1011492. doi:10.1371/journal.ppat.1011492 37459363 PMC10374047

[143] Sanjana NE, Shalem O, Zhang F. 2014. Improved vectors and genome-wide libraries for CRISPR screening. Nat Methods 11:783–784. doi:10.1038/nmeth.3047 25075903 PMC4486245

