# Supplementary material for: Single-cell delineation of strain-specific HIV-1 Vif activities using dual reporter sensor cells and live cell imaging
Source: J Virol. 2025 Feb 25;99(3):e01579-24. doi: 10.1128/jvi.01579-24 (PMC11915839; doi:10.1128/jvi.01579-24)
Supplement: Supplementa legends — Legends for Movies S1 to S3. [file jvi.01579-24-s0001.docx]

**Supplementary Movie Figure Legends**

**Movie S1.** ***DRSC activation***. Time-lapse series of a DRSC being infected by a HIV-CFP virus at an MOI of 1, as shown in Figure 1. Movie shows onset of CFP expression (cyan, indicating infection), followed by loss of YFP-A3G (yellow) and activation of Gag-mCherry expression (magenta). (Right) YFP channel (A3G), mCherry channel (Gag), and CFP channel (HIV-1) shown separately. (Left) Merge of YFP, mCh, and CFP channels. Images were acquired every hour over a 20h time course using a 20x objective.

**Movie S2.** ***DRSCs infected at high or low MOI.*** Time-lapse series of DRSCs infected under low (left, MOI = 1) or high (right, MOI = 3) MOI conditions, with these data corresponding to Figure 2. High MOI infection yielded faster YFP-A3G (yellow) degradation as well as greater expression of Gag-mCherry (magenta) compared to the low MOI infection. Bottom panels show separated YFP and mCherry channels. Top panels show the merged time-lapse of images. Images were acquired every hour over a 20h time course using a 20x objective.

**Movie S3**. ***B3 and C1 Vif demonstrate defective anti-A3G activity.*** 20h time-lapse series showing DRSCs infected with the indicated chimeric Vif viruses, corresponding to Figure 3. From left to right, NL4-3, Vif(-), B3, C1, and high MOI B3 Vif (MOI = 3) infections are shown. Top panel displays the merge of the YFP-A3G and Gag-mCh channels, while the bottom panel shows the YFP channel only. The arrows in the “B3 High” movie point to sites of Gag-mCherry at bud sites accumulating with YFP-A3G, consistent with the delayed kinetics of YFP-A3G degradation observed for B3 Vif. Images were acquired every hour over a 20h time course using a 20x objective.
